# Evaluating the origin and virulence of a *Helicobacter pylori cagA*-positive strain isolated from a non-human primate

**DOI:** 10.1038/s41598-018-34425-4

**Published:** 2018-10-29

**Authors:** Kana Hashi, Chihiro Imai, Koji Yahara, Kamrunnesa Tahmina, Takeru Hayashi, Takeshi Azuma, Takako Miyabe-Nishiwaki, Hideyuki Sato, Masao Matsuoka, Sachi Niimi, Munehiro Okamoto, Masanori Hatakeyama

**Affiliations:** 1 Division of Microbiology, Graduate School of Medicine, The University of Tokyo, Tokyo, 113-0033 Japan; 2 Antimicrobial Resistance Research Center, National Institute of Infectious Diseases, Higashimurayama, Tokyo, 189-0002 Japan; 3 Department of Gastroenterology, Kobe University Graduate School of Medicine, Kobe, 650-0017 Japan; 4 Primate Research Institute, Kyoto University, Aichi, 484-8506 Japan; 5 Division of Stem Cell Therapy, Center for Stem Cell Biology and Regenerative Medicine, Institute of Medical Science, The University of Tokyo, Tokyo, 108-8639 Japan; 6 Institute for Frontier Life and Medical Sciences, Kyoto University, Kyoto, 606-8507 Japan

## Abstract

*Helicobacter pylori cagA*-positive strains are critically involved in the development of gastric cancer. Upon delivery into gastric epithelial cells via type IV secretion, the *cagA*-encoded CagA interacts with and thereby perturbs the pro-oncogenic phosphatase SHP2 and the polarity-regulating kinase PAR1b via the tyrosine-phosphorylated EPIYA-C/D segment and the CM sequence, respectively. Importantly, sequences spanning these binding regions exhibit variations among CagA proteins, which influence the pathobiological/oncogenic potential of individual CagA. Here we isolated an *H*. *pylori* strain (Hp_TH2099) naturally infecting the stomach of a housed macaque, indicating a zoonotic feature of *H*. *pylori* infection. Whole genome sequence analysis revealed that Hp_TH2099 belongs to the hpAsia2 cluster and possesses ABC-type Western CagA, which contains hitherto unreported variations in both EPIYA-C and CM sequences. The CM variations almost totally abolished PAR1b binding. Whereas pTyr + 5 variation in the EPIYA-C segment potentiated SHP2-binding affinity, pTyr-2 variation dampened CagA tyrosine phosphorylation and thus impeded CagA-SHP2 complex formation. As opposed to the *H*. *pylori* standard strain, infection of mouse ES cell-derived gastric organoids with Hp_TH2099 failed to elicit CagA-dependent epithelial destruction. Thus, the macaque-isolated *H*. *pylori* showed low virulence due to attenuated CagA activity through multiple substitutions in the sequences involved in binding with SHP2 and PAR1b.

## Introduction

*Helicobacter pylori*, a micro-aerophilic spiral-shaped bacterium colonizing the human stomach, is a major etiologic agent of atrophic gastritis and peptic ulcers^1^. Epidemiological studies have also shown that chronic infection with *H*. *pylori* is critically associated with the development of gastric cancer^2–4^. Individually isolated *H*. *pylori* is subdivided into *cagA*-positive and -negative strains based on the presence or absence of the *cag* pathogenicity island (*cag*PAI), a genomic region that encodes a bacterial type IV secretion system (TFSS) and its effector CagA^5^. Accumulating evidence has shown that infection with *cagA*-positive strains, which inject CagA into gastric epithelial cells via the TFSS, plays a critical role in gastric carcinogenesis^6,7^.

The C-terminal region of CagA contains multiple Glu-Pro-Ile-Tyr-Ala (EPIYA) motifs, which serve as sites for tyrosine phosphorylation by Src-family kinases (SFKs) or c-Abl^8^. The EPIYA-repeat region is composed of various combinations of four distinct EPIYA segments, EPIYA-A, -B, -C, and -D, which are defined by the amino acid sequence surrounding each of the EPIYA motifs^9^. The *cagA* genomic sequence encoding the EPIYA-repeat region is frequently recombined and thereby creates a structural polymorphism that enables classification of individual CagA into several subtypes^9,10^. The two major CagA subtypes are Western CagA and East Asian CagA. *H*. *pylori cagA*-positive strains circulating all over the world, except for east Asian countries such Japan, China and Korea, carry Western CagA containing EPIYA-A, EPIYA-B, and a variable number of tandem-repeated EPIYA-C segments (usually 1~3 times), whereas the vast majority of *cagA*-positive strains circulating in East Asia carry East Asian CagA containing EPIYA-A, EPIYA-B, and EPIYA-D segments. In addition, CagA possesses a 16-amino-acid sequence termed the CagA multimerization (CM) sequence^11^. The predominant Western CagA contains a single EPIYA-C segment (ABC-type CagA) and thereby possesses two CM sequences, one comprising the N-terminal region of EPIYA-C and the other located immediately downstream of EPIYA-C. In contrast, prevalent East Asian CagA (ABD-type CagA) contains only a single CM sequence immediately downstream of the EPIYA-D segment. The CM sequences are similar but not identical in Western CagA and East Asian CagA. CagA binds to SHP2 via the tyrosine-phosphorylated EPIYA-C or EPIYA-D segment^12,13^. SHP2 is a pro-oncogenic/pro-mitogenic phosphatase that stimulates cell proliferation and cell motility, and indeed gain-of-function SHP2 mutants have been found in a variety of human malignancies^14^. Upon binding, CagA deregulates the catalytic activity of SHP2 to promote pro-oncogenic cellular events. Additionally, CagA interacts with the polarity-regulating kinase PAR1b (also known as MARK2) via the CM sequence^15^. The CagA-PAR1b interaction inhibits PAR1b kinase activity and thereby causes junctional and polarity defects. Sequence polymorphisms in the EPIYA and CM sequences influence the binding strength of CagA with SHP2 and PAR1b^16–19^, respectively, which underscores the differential pathobiological activity of individual CagA that contributes to gastric carcinogenesis^19^. Indeed, oncogenic potential of *H*. *pylori* CagA *in vivo* has been demonstrated in CagA-transgenic mice^20^.

Rodents have been extensively used as *in vivo* models for studying the virulence of *H*. *pylori*. However, *H*. *pylori* strains adapted in rodents often lose the functional TFSS and thus fail to deliver CagA^21,22^. *H*. *pylori* can also infect non-human primates, and macaques have been used as an experimental model for *H*. *pylori* infection^23–26^. Again, however, studies with non-human primates are time-consuming, tedious, labor-intensive, and extremely expensive in cost, making it difficult to evaluate the degree of virulence for individual *cagA*-positive strains using non-human primates. It has recently become possible to generate organ-specific three-dimensional cultures of cells, known as “organoids”^27^. Gastric organoids generated from isolated gastric mucosa are spheres of epithelial cell layer with the lumen inside, which can be infected with *H*. *pylori*^28–30^. Gastric organoids have also been developed from embryonic stem (ES) cells^31,32^. Organoids derived from ES cells may be more appropriate than organoids derived from gastrointestinal crypts for studying host-pathogen interaction as they comprise three distinct components: epithelial cell layer, mesenchymal cells, and lamina muscularis mucosae.

In this work, we isolated an *H*. *pylori cagA*-positive strain termed Hp_TH2099, which belongs to the hpAsia2 cluster, from a housed macaque and found that the Hp_TH2099 strain carries a Western CagA protein that possesses heretofore unreported substitutions in the EPIYA-repeat region, which affect SHP2 and PAR1b binding. We also evaluated the virulence of the Hp_TH2099 strain using gastric organoids derived from mouse ES cells.

## Results

### Isolation and genomic analysis of macaque-derived *H*. *pylori*

Previous studies showed natural infection with *H*. *pylori cagA*-positive strains in captive rhesus macaques^23–25^. To investigate the pathobiological properties of macaque *H*. *pylori*-derived CagA, we sought to isolate *cagA*-positive *H*. *pylori* from the stomachs of macaques individually housed at the Primate Research Institute, Kyoto University (KUPRI). Since the EPIYA-repeat region is crucial for CagA activity, universal primers amplifying a *cagA* gene segment encoding the EPIYA-repeat region were constructed on the basis of currently available *cagA* sequences registered in NCBI. Using these primers, a DNA fragment with approximately 1,000 base pairs (bps) was amplified from DNA purified from gastric juice of three rhesus macaques (ID: Mm1689, Mm1874, Mm1887) that had been housed together in childhood (Supplementary Fig. S1). Since *H*. *pylori* is the only bacterium known to carry *cagA*, the DNA fragment was most likely from macaque-derived *H*. *pylori*. To isolate *H*. *pylori*, gastric juice was collected from several distinct macaque species housed in KUPRI (the above-stated 3 rhesus macaques, 1 Taiwanese macaque, 5 Japanese macaques), mixed with a *Helicobacter* selective medium, stored at 4 °C, and then plated within 48 hours. The plates were incubated at 37 °C in 5% CO_2_ in an incubator for 3–7 days until colonies grew. Colony direct PCR was then performed using a *cagA* primer set that specifically amplifies a ~750-bp *cagA* fragment. The results of PCR revealed the presence of the *cagA* gene in several bacterial colonies isolated from a Japanese macaque (ID: TH2099) (Supplementary Fig. S2). No *cagA* fragment was PCR-amplified from bacterial colonies isolated from other macaques, including the 3 rhesus macaques. This was most probably because the gastric samples had been preserved under non-optimal conditions for *H*. *pylori* survival. The *cagA*-positive bacterium isolated from the macaque (hereafter termed Hp_TH2099) showed microbiological properties that are consistent with *H*. *pylori* species (Supplementary Fig. S3).

**Figure 1:**
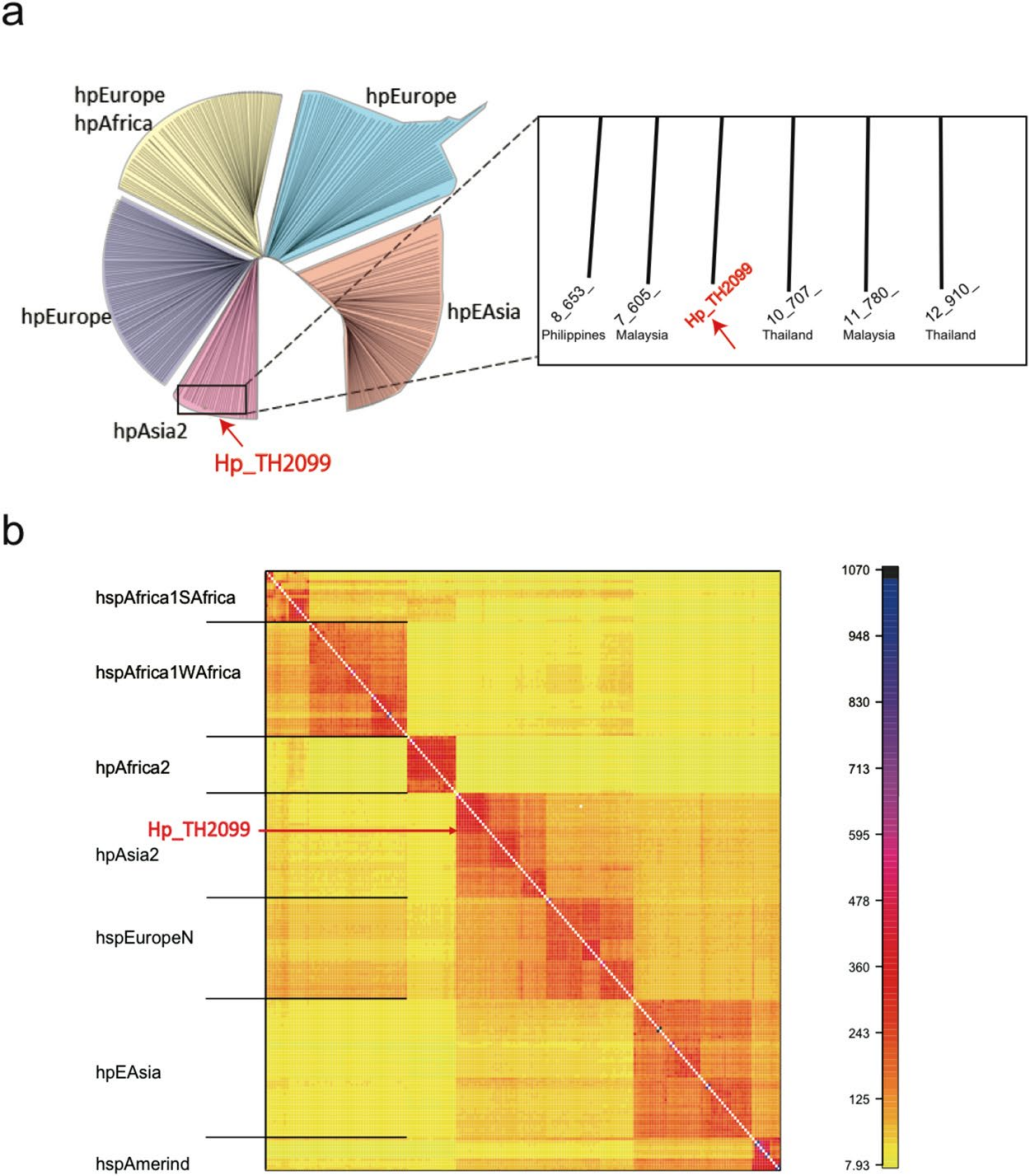
Isolation of *cagA*-positive *H*. *pylori* from macaque stomach. (**a**) Molecular phylogenetic tree based on MLST analysis. Seven *H*. *pylori* genes (*atpA*, *efp*, *mutY*, *ppa*, *trpC*, *urel*, *yphC*) were used to perform MLST analysis (left). Magnified view of the phylogenetic tree in the vicinity of Hp_TH2099 (right). (**b**) fineSTRUCTURE analysis of the Hp_TH2099 genome. The color of each cell of the matrix indicates the expected number of DNA chunks imported from a donor genome (column) to a recipient genome (row). The boundaries between named populations are marked with lines. Detailed information on *H*. *pylori* isolates used in this analysis is shown in Supplementary Table S1.

To find the nearest phylogenetic neighbor of Hp_TH2099, whole genome analysis was conducted. Using the genome data, a phylogenetic tree was drawn on the basis of Multi Locus Sequencing Typing (MLST) and the Hp_TH2099 strain fitted within the *H*. *pylori* hpAsia2 cluster^33^ (Fig. 1a). Population structure analysis at a finer scale, called fineSTRUCTURE^34^, was also performed using the whole genome sequences and the results consolidated that the isolated *H*. *pylori* strain was within the hpAsia2 cluster and more specifically in a subgroup consisting of hpAsia2 strains isolated from humans mostly in Malaysia^35^ (Fig. 1b, Supplementary Table S1).

### Analysis of the Hp_TH2099 *cagA* gene and its encoded CagA protein

**Figure 2:**
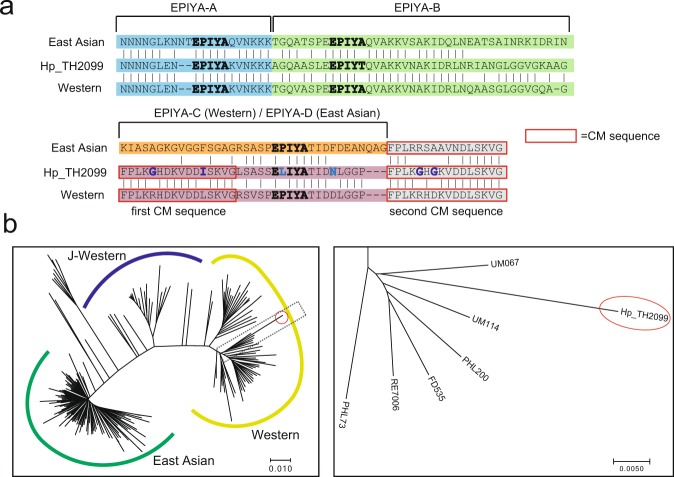
Sequence analysis of Hp_TH2099 CagA. (**a**) Amino acid sequences for the EPIYA-repeat regions of canonical East Asian CagA, Hp_TH2099 CagA, and canonical Western CagA were aligned using Clustal W. Amino acid variations in Hp_TH2099 CagA are indicated in dark blue and light blue bold letters. (**b**) Phylogenetic tree based on the nucleotide sequence of the Hp_TH2099 *cagA* gene (shown in a red circle) with 199 full-length *cagA* genes extracted from the GenBank database (left). Positions of East Asian *cagA*, Western *cagA* and J-Western *cagA* were indicated in green line, yellow line, and blue line, respectively. Magnified view of the black dotted box in left figure (right). Hp_TH2099 and other closely related *cagA* genes were shown. The tree is drawn to scale with branch lengths in the same units as those of the evolutionary distances used to infer the phylogenetic tree. The evolutionary distance was computed in the units of the number of base substitutions per site.

The genome sequence analysis revealed that the Hp_TH2099 genome possesses *cag*PAI that codes for TFSS (Table 1). The *cag*PAI also contained the Hp_TH2099 *cagA* gene that comprises 3,450 bps in length, encoding a CagA protein with 1,150 amino acid residues (Supplementary Fig. S4). The amino acid sequence of the EPIYA-repeat region in Hp_TH2099 CagA was then aligned with those of Western CagA and East Asian CagA. As a result, Hp_TH2099 CagA was found to contain the EPIYA-A segment, EPIYA-B segment, and EPIYA-C segment in that order (Fig. 2a), indicating that it belongs to ABC-type Western CagA. A phylogenetic tree drawn using previously reported full-length *cagA* sequences consolidated that Hp_TH2099 *cagA* was a member of the Western *cagA* group (Fig. 2b, left). There is a minor subtype of Western *cagA*, designated J-Western *cagA*, that was originally identified in *H*. *pylori* strains isolated from Okinawa islands, Japan and has subsequently been found in Southeast Asia, Europe, and North America^36,37^. The Hp_TH2099 *cagA* belonged to the major Western *cagA* group but not the J-Western *cagA* group and was most closely related to the *cagA* gene carried by the UM067 strain (Fig. 2c, right), which was isolated from a peptic ulcer patient in Malaysia^35^.

**Table 1:** Alignment of Type IV secretion system in Hp_TH2099.

gene ID	presence (1) or absence (0) in TH2099_contigs	% identity	% aligned length
HP0519	1	92.16	102.89
HP0520	0	88.46	7.47
HP0522	1	95.09	100
HP0523	1	92.16	100
HP0524	1	95.55	100
HP0525	1	97.68	100
HP0526	1	98.67	100
HP0527	0	96.88	28.86
HP0528	1	97.64	100
HP0529	1	97.01	100
HP0530	1	97.36	100
HP0531	0	76.36	8.37
HP0532	1	98.56	57.77
HP0533	1	95.56	100
HP0534	0	97.51	34.01
HP0535	0	100	4.46
HP0536	0	90.48	6.09
HP0537	0	82.86	3.09
HP0538	1	97.72	100
HP0539	1	95.18	95.94
HP0540	1	95.81	100
HP0541	1	96.59	100
HP0542	1	97.67	100
HP0543	1	96.41	100
HP0544	1	97.96	91.43
HP0545	1	96.15	100
HP0546	1	94.25	100
HP0547	1	92.14	100.08
HP0549	1	95.7	100

The *cag*PAI gene sequences of Hp_TH2099 were extracted from whole genome sequencing data and were aligned with *cag*PAI genes of 26695 strain by BLAST software. A BLAST matches with greater than 70% identity and greater than 50% of the locus length were indicated as “Presence”.

The EPIYA-repeat region of Hp_TH2099 CagA contained hitherto unreported variations in amino acid residues that appear to influence CagA binding with SHP2 and PAR1b. Specifically, the two CM sequences in Hp_TH2099 CagA had several amino acid alterations, Arg-to-Gly (residue 915) and Leu-to-Ile (residue 922) substitutions in the first (proximal) CM sequence and Arg-to-Gly (residue 949) and Asp-to-Gly (residue 951) substitutions in the second (distal) CM sequence, compared to the canonical CM sequence (Fig. 2a)^11,18^. All of these alterations occurred at residues that may be crucial for PAR1b binding^38^ and there was no previous report for the Western CagA variant that possesses Gly instead of Arg at the N-terminal fifth position in both the proximal and distal CM sequences. Hp_TH2099 CagA also contained novel amino acid alterations within the EPIYA-C segment: the -2 position (residue 933) from the tyrosine phosphorylation site in the EPIYA motif (pTyr) was Leu instead of 100% conserved Pro in other CagA proteins. Additionally, the pTyr + 5 position (residue 940), which plays a pivotal role in determining the SHP2-binding affinity of CagA^9,39^, was changed from highly conserved Asp to Asn in Hp_TH2099 CagA (Fig. 2a). The entire *cagA* genes that were amplified by PCR from the gastric DNA samples of the rhesus macaques (Mm1874 and Mm1887) were highly related to Hp_TH2099 *cagA* and the sequences encoding the EPIYA-C segment and the CM sequence were 100% identical to those of Hp_TH2099 *cagA* (Supplementary Fig. S5). The finding indicated that Hp_TH2099-like *H*. *pylori* strains were possibly widespread among distinct macaque species housed in KUPRI. Also notably, whereas Hp_TH2099 CagA was most closely related to UM067 CagA isolated from a Malaysian, the above-described substitutions in the EPIYA-C and CM sequences were not present in UM067 CagA.

### PAR1b-binding activity of Hp_TH2099 CagA

**Figure 3:**
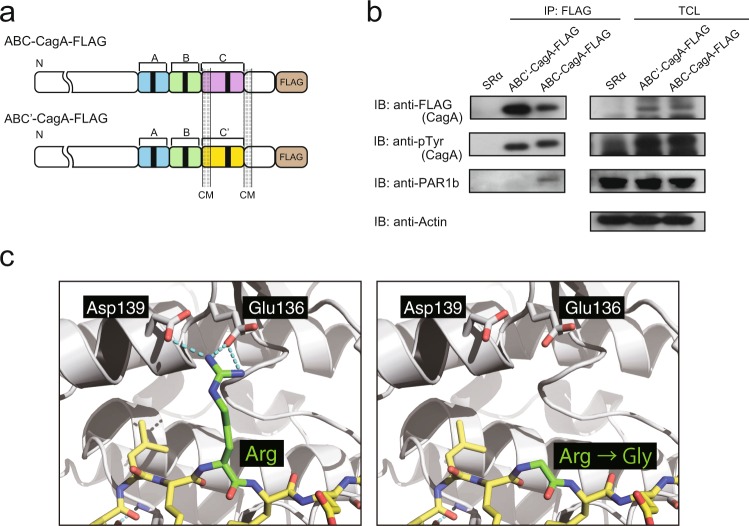
PAR1b-binding activity of Hp_TH2099 CagA. (**a**) A schematic view of ABC-CagA and ABC’-CagA proteins. ABC-CagA contains the canonical EPIYA-A, EPIYA-B and EPIYA-C segments as well as canonical CM sequences. ABC’-CagA contains canonical EPIYA-A and EPIYA-B segments followed by a variant EPIYA-C segment (EPIYA-C’) and a variant CM sequence derived from Hp_TH2099 CagA. (**b**) AGS cells were transiently transfected with an ABC’-CagA-FLAG vector, an ABC-CagA-FLAG vector, or an empty control vector (SRα). Cell lysates were immunoprecipitated with an anti-FLAG antibody and the immunoprecipitates were subjected to immunoblotting analysis with the indicated antibodies. TCL: total cell lysate. IP: immunoprecipitate. Full-length blots are presented in Supplementary Figure S12. (**c**) Structural prediction of the Hp_TH2099 CagA-PAR1b interaction was carried out by using MacPyMOL v1.8.6.2 based on the crystal structure of Western CagA-PAR1b complex (PDB ID: 3IEC)^38^. Interface structure of the Western CagA-PAR1b complex is shown with yellow stick and grey ribbon models for CagA and PAR1b, respectively (left). The conserved Arg residue in the CM sequence of Western CagA was replaced with Gly as observed in Hp_TH2099 CagA (right). Cyan dashed lines indicate polar contacts.

To investigate the function of Hp_TH2099 CagA, we generated an expression vector for canonical ABC-type Western CagA with C-terminal FLAG tag (ABC-CagA-FLAG) or its derivative (ABC’-CagA-FLAG) in which the EPIYA-C segment and the second CM sequence were replaced by those derived from Hp_TH2099 CagA (Fig. 3a). The construct ensured that the sequence structures other than those for the EPIYA-C segment and the second CM sequence were identical in ABC-CagA and ABC’-CagA. The ABC-CagA-FLAG or ABC’-CagA-FLAG vector was transiently transfected into AGS cells and the cell lysates were immunoprecipitated with an anti-FLAG antibody. The results of the experiment revealed that, in contrast to ABC-CagA, ABC’-CagA exhibited little or no binding with PAR1b (Fig. 3b). A previous crystal analysis of the CagA-PAR1b complex showed that the interaction is stabilized via multiple salt bridges between the Arg residue, located at the + 5 position from the N-terminus in the CM sequence, and Glu136 and Asp139 of PAR1b^38^. The same study also showed that the artificial mutation of the Arg residue in the CM sequence to Gly markedly reduces PAR1b binding. Since Hp_TH2099 CagA naturally contains Gly instead of Arg (residues 915 and 949) at the + 5 position in both of the CM sequences, we generated structural models to elucidate the mechanism by which Hp_TH2099 CagA lost PAR1b-binding activity. The models predicted that Arg-to-Gly replacement in the CM sequences abolishes the Arg-mediated electrostatic interactions and thereby dampens the PAR1b binding (Fig. 3c). Since Leu at the 12th position from the N-terminus in the canonical CM sequence is another residue substantially involved in CagA-PAR1b binding^38^, we also generated a structural model with focus on the contribution of the Leu residue. Crystal structure showed that the main chain of Leu was fixed in the deep hydrophobic pocket via hydrogen bond formation with Phe209 of PAR1b (Supplementary Fig. S6). The hydrophobic pocket on PAR1b provides a precise space for two δ-methyl groups of Leu but less space for the γ-methyl group of Ile. It is therefore possible that the Leu-to-Ile substitution at the position impedes the interaction of Hp_TH2099 CagA with PAR1b by inducing steric hindrance that occurs due to the presence of the proximal γ-methyl group branched from the β-carbon in Ile.

### SHP2-binding activity of Hp_TH2099 CagA

**Figure 4:**
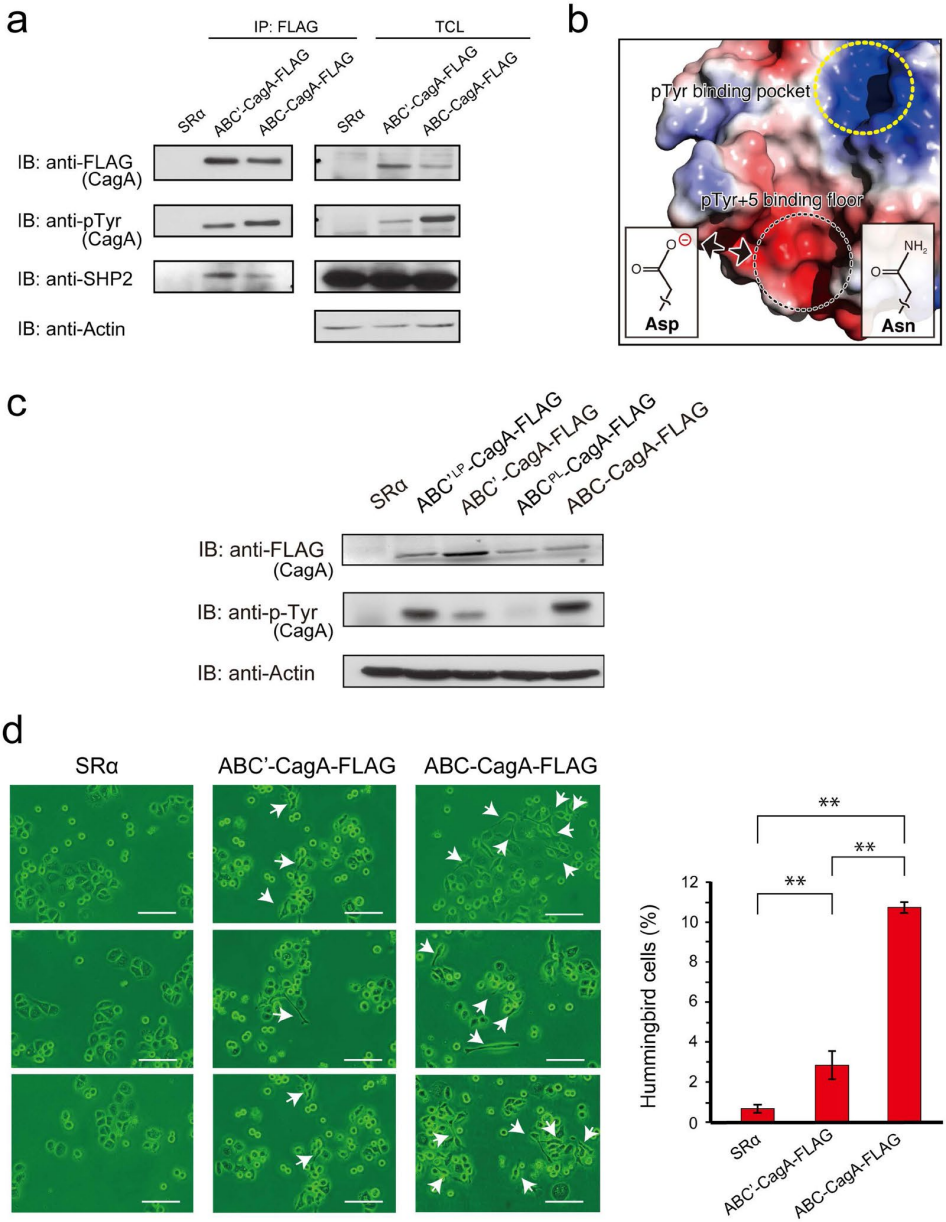
SHP2-binding activity of Hp_TH2099 CagA. (**a**) AGS cells were transiently transfected with an ABC’-CagA-FLAG vector, an ABC-CagA-FLAG vector, or an empty control vector (SRα). Cell lysates were immunoprecipitated with an anti-FLAG antibody and the immunoprecipitates were subjected to immunoblotting analysis with the indicated antibodies. TCL: total cell lysate. IP: immunoprecipitate. Full-length blots are presented in Supplementary Figure S12. (**b**) CagA-binding interface of the SHP2 N-SH2 domain based on the data described previously^39^. Red-to-blue represents acidic-to-basic on the surface of the SHP2 N-SH2 domain. A dot circle in yellow indicates the region that interacts with the pTyr + 5 residue of the EPIYA-C segment. A dot circle in black indicates the region corresponding to the pTyr-binding pocket. (**c**) AGS cells were transiently transfected with an ABC’-CagA-FLAG vector, an ABC’^LP^-CagA-FLAG vector, an ABC-CagA-FLAG vector, an ABC^PL^-CagA-FLAG vector, or an empty control vector (SRα). In the ABC’^LP^-CagA-FLAG mutant, the ELIYA sequence in the EPIYA-C segment of ABC’-CagA-FLAG was replaced by the EPIYA sequence. In the ABC^PL^-CagA-FLAG mutant, the EPIYA sequence in the EPIYA-C segment of ABC-CagA-FLAG was replaced by the ELIYA sequence. Cell lysates were immunoprecipitated with an anti-FLAG antibody and the immunoprecipitates were subjected to immunoblotting analysis with the indicated antibodies. Full-length blots are presented in Supplementary Figure S12. (**d**) The morphology of AGS cells transfected with an ABC’-CagA-FLAG or an ABC-CagA-FLAG vector was examined under a microscope at 17 hours after transfection. Scale bars: 100 µm (left). Percentages of cells showing the hummingbird phenotype were calculated and statistically analyzed by using Student’s *t*-test. Error bars represent ± S.D. (n = 3), **p < 0.01. (right).

Besides the CM sequences, Hp_TH2099 CagA also contained two unique amino acid substitutions in the EPIYA-C segment, one at the pTyr-2 position and the other at the pTyr + 5 position (Fig. 2a). It has been reported that the pTyr + 5 residue in the EPIYA-C or EPIYA-D segment is a critical residue that determines the binding affinity to SHP2^9,39^. The pTyr + 5 residue of EPIYA-C in Hp_TH2099 CagA is Asn instead of the highly conserved Asp in Western CagA. To compare the SHP2-binding activity of Hp_TH2099-derived EPIYA-C with that of canonical EPIYA-C, AGS cells were transiently transfected with an ABC-CagA-FLAG or ABC’-CagA-FLAG vector and the cell lysates were used in a co-immunoprecipitation experiment. As a result, Hp_TH2099-derived EPIYA-C bound SHP2 more efficiently than canonical EPIYA-C did (Fig. 4a). Thus, Asp-to-Asn substitution at pTyr + 5 potentiated the SHP2-binding activity of CagA. The conclusion was supported by the binding model between the EPIYA-C segment and the SHP2 N-SH2 domain reported previously^39^. The model predicted that the N-SH2 floor that interacts with the pTyr + 5 residue of the EPIYA-C segment is highly acidic and thereby generates a propulsive electrostatic force with the acidic residues such as Asp (Fig. 4b, lower dotted circle). In other words, Asp at pTyr + 5 in canonical EPIYA-C was inhibitory rather than stimulatory in terms of SHP2 binding and Asp-to-Asn replacement at pTyr + 5 was considered to potentiate the binding in the absence of electrical repulsion. At the same time, however, we also noticed that the Hp_TH2099 EPIYA-C segment underwent less tyrosine phosphorylation than did canonical EPIYA-C despite comparable levels of expression (Fig. 4a). We hypothesized that the reduced tyrosine phosphorylation was due to Pro-to-Leu substitution at pTyr-2 in the Hp_TH2099 EPIYA-C segment, which disrupts the EPIYA tyrosine-phosphorylation motif recognized by SFKs and c-Abl. The idea was supported by the finding that Leu-to-Pro replacement in the ELIYA sequence elevated the tyrosine phosphorylation level of ABC’-CagA whereas Pro-to-Leu replacement in the EPIYA sequence decreased the tyrosine phosphorylation level of ABC-CagA (Fig. 4c). Taken these together, the unique amino acid alterations in the EPIYA-C segment of Hp_TH2099 CagA have both positive and negative effects on SHP2 binding and the net result of these substitutions was reduced SHP2 binding compared to the canonical EPIYA-C segment (Fig. 4a). Since deregulation of SHP2 by CagA is indispensable for induction of the elongated cell shape known as the hummingbird phenotype^12,39^, we also compared the magnitude of hummingbird phenotype induction by ABC-CagA and ABC’-CagA in AGS human gastric epithelial cells. Whereas the expression level of ABC-CagA was greater than that of ABC-CagA in this transfection experiment, ABC’-CagA was substantially less active than ABC-CagA in inducing the hummingbird phenotype despite greater SHP2 binding (Fig. 4d, Supplementary Fig. S7). We also confirmed that ABC’-CagA was less tyrosine-phosphorylated than ABC-CagA was (Supplementary Fig. S7). Thus, the attenuated morphogenetic activity of CagA was due to (1) reduced SHP2 binding and (2) loss of PAR1b binding that enhances the CagA-SHP2-mediated induction of the hummingbird phenotype^40,41^.

### Generation of organoids with a stomach tissue-like property from mouse ES cells

Infection of monkey COS7 cells with the Hp_TH2099 strain induced IL-8 mRNA, indicating that Hp_TH2099 possesses TFSS that functionally interacts with monkey cells (Supplementary Fig. S8). However, since there were substantial variations in the sequences of certain core proteins of TFSS in the Hp_TH2099 strain (Table 1), we generated an isogenic Hp_TH2099 strain lacking the *cagA* gene (Supplementary Fig. S9a and b), the product of which could potentially influence IL-8 mRNA induction at later phases of *H*. *pylori* infetion^42^. Upon infection of AGS cells with the *cagA*-negative isogenic strain, which did not produce CagA and thus failed to deliver CagA and subsequent induction of the hummingbird phenotype, IL-8 mRNA was induced to the level that was comparable to those induced by infection with wild-type Hp_TH2099 or the G27 standard *H*. *pylori* strain (Supplementary Fig. S9c). The IL-8 mRNA induction in AGS cells was totally dependent on the functional TFSS as G27 lacking the *cag*PAI segment (G27*Δcag*PAI) failed to do so. These observations provided compelling evidence that the macaca-isolated *H*. *pylori* strain possesses the functional TFSS despite the presence of sequence diversity in their protein components.

Upon delivery into AGS cells, Hp_TH2099 CagA gave rise to induction of the hummingbird phenotype (Supplementary Fig. S10). Whereas the hummingbird phenotype has been thought to correlate with the virulence of individual CagA^9,13,17,19^, little is known about the direct impact of *H*. *pylori*-delivered CagA on the stomach mucosa. We therefore sought to establish an *ex vivo* system that can evaluate the effect of *cagA*-positive *H*. *pylori* infection on the epithelia by using gastric organoids derived from ES cells (Supplementary Fig. S11a). After confirming the stemness of the mouse ES cell line EB3 under feeder-free conditions^43^ (Supplementary Fig. S11b), the cells were cultured in an Iscove’s modified Dulbecco’s medium (IMDM)-based medium for 4 days to form embryonic bodies (EBs). The obtained EBs were then cultured in 24-well gelatin-coated dishes with IMDM-based medium for additional 4 days as previously reported^44^. Those EBs were found to be positive for definitive endoderm markers, E-cadherin, Sox17, and Goosecoid^45^ (Supplementary Fig. S11c). Human ES cell-derived definitive endoderm has been shown to differentiate into a gastric antral-like organoid through a posterior foregut spheroid when cultured in RPMI-based FGF4-noggin medium^31^. Accordingly, the mouse ES-derived EBs were cultured in RPMI 1640 medium containing 2% fetal bovine serum (FBS), 100 ng/ml mouse fibroblast growth factor 4 (FGF4), 50 ng/ml mouse noggin, and 3 µM CHIR-99021, a selective GSK3 inhibitor. Since retinoic acid (RA) is essential for differentiation into the posterior foregut^31^, 2 mM RA was added to the medium 48 hours after the onset of RPMI-based culture. Three days later, floating spheroids were observed (Supplementary Fig. S10d). Immunostaining analysis revealed that the spheroids were positive for the posterior foregut markers, Hnf1b and Sox2^31^ (Supplementary Fig. S11d). The spheroids were then picked up and cultured in Matrigel with advanced DMEM/F12-based medium as previously described^31^. After 26 days of differentiation from ES cells, organoids of about 1 mm in diameter had developed. The organoids could be further kept in the matrigel by adding 100 ng/ml EGF after day 26. By day 40, the diameters of organoids had become approximately 1.5 mm (Supplementary Fig. 12a). HE staining and PAS staining indicated that the organoids consisted of an epithelial layer producing mucus and stromal components (Supplementary Fig. 12b). To determine whether the mouse ES cell-derived organoids had acquired the properties of gastric tissue, RNA microarray analysis was performed using mRNAs isolated from the organoids on day 26. The results of hierarchical clustering analysis showed that the RNA expression patterns of the organoids were more similar to those of adult mouse stomach tissue than fetal mouse stomach tissue Supplementary Fig. 12c). Consistently, an immunostaining experiment revealed that the organoids had an inner layer of E-cadherin-positive epithelial cells that produced mucin 5ac and mucin 6 and expressed the pyloric gland-specific marker gastrin (Supplementary Fig. 12d). Also, there was an outer layer of vimentin-expressing mesenchymal-like cells as well as myofibroblast-like cells expressing α-SMA (Supplementary Fig. 12e). These results indicated that the mouse ES cell-derived organoids acquired stomach tissue-like properties.

### Impact of *H*. *pylori* infection on ES cell-derived organoids

**Figure 5:**
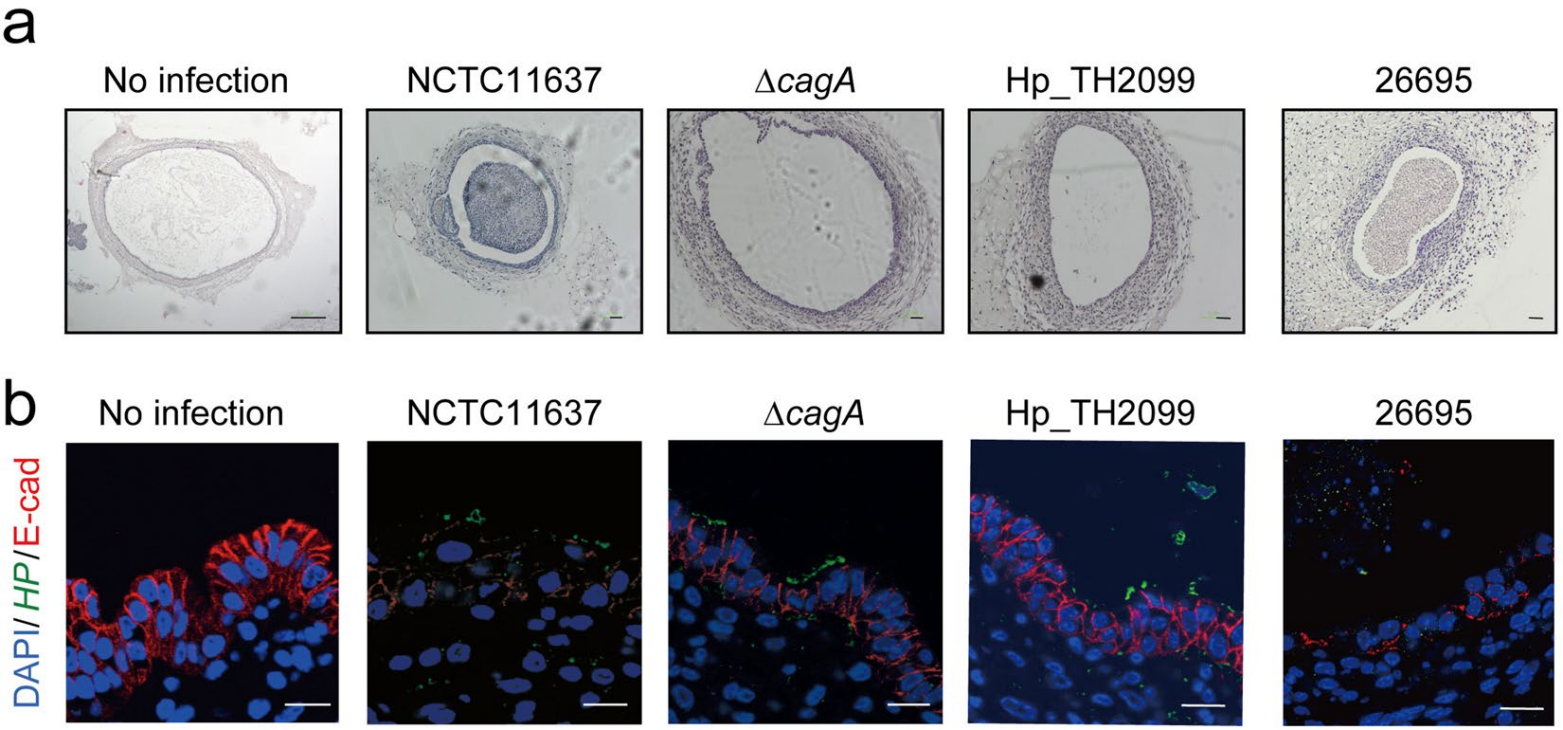
Impact of *H*. *pylori* infection on ES cell-derived gastric organoids. (**a**) HE staining of ES-cell derived gastric organoids infected with *H*. *pylori* for 12 hours. Scale bar: 200 µm. (**b**) Immunostaining of gastric organoids infected with *H*. *pylori* for 12 hours. “No infection” indicates that the organoid was injected with bacteria-free Brucella Broth. *HP*, *H*. *pylori*; E-cad, E-cadherin. Scale bar: 10 µm.

To evaluate the influence of *cagA*-positive *H*. *pylori* infection on the ES-cell derived gastric organoids, the NCTC11637 standard strain or an isogenic *cagA-*negative strain (NCTC11637Δ*cagA*) was injected into the lumens of the organoids (at day 40 of culture). Twelve hours later, the *H*. *pylori*-injected organoids were paraffin-embedded and were subjected to histological analysis using microscopy. In the organoids injected with the NCTC11637 strain, a large numbers of epithelial cells were cast off from the luminal surface, forming an aggregation of dead cells in the lumen (Fig. 5a). Furthermore, membrane staining of E-cadherin was dramatically reduced in the resting epithelial layer of NCTC11637-injected organoids (Fig. 5b). On the other hand, the organoids infected with the isogenic Δ*cagA* strain showed no epithelial damage or alteration of E-cadherin distribution at the lateral membrane (Fig. 5a and b), indicating that the epithelial layer in the organoids was highly sensitive to the bacterially injected CagA. Given this, we next injected the 26695 standard strain, which contains canonical ABC-type Western CagA, or the Hp_TH2099 strain into the organoids. Like the NCTC11637 strain, infection with the 26695 strain caused extensive disruption of the epithelial layer. In contrast, infection with the Hp_TH2099 strain caused no structural disorganization in the epithelial layer of the gastric organoids (Fig. 5a and b). From these observations, the macaque-derived *H*. *pylori* strain was considered to have attenuated virulence in terms of CagA-mediated epithelial damage.

## Discussion

The natural habitat of *H*. *pylori* is the human stomach mucosa. However, *H*. *pylori* can experimentally infect animals such as mice, gerbils, and macaques in a laboratory. Furthermore, some macaques reared in captivity are naturally infected with *H*. *pylori*^22–24^. The observations indicate that non-human primates are also natural hosts of *H*. *pylori*, though it is unclear whether macaques carry *H*. *pylori* as a natural reservoir in wild life or whether *H*. *pylori* is transmitted from humans to macaques after captivity. In this study, we isolated an *H*. *pylori cagA*-positive strain, termed Hp_TH2099, from the stomach of a housed Japanese macaque, which was born in KUPRI. The results of MLST analysis revealed that the Hp_TH2099 strain belongs to the group hpAsia2, which consists of *H*. *pylori* strains predominantly distributed in India and Southeast Asian countries such as Malaysia, Thailand, Philippines and Bangladesh^35^. Also, Hp_TH2099 possesses a *cagA* gene that encodes an ABC-type Western CagA. Since virtually all of the *H*. *pylori* strains isolated from Japanese people carry East Asian CagA and since most of the staff members of KUPRI have been Japanese, it is reasonable to speculate that the Hp_TH2099 strain originated from *H*. *pylori* infecting the ancestors of the macaques caught in India/Southeast Asia before they were brought to KUPRI. The origin of Hp_TH2099 *H*. *pylori* may then have expanded across different macaque species, including Japanese macaques, through natural infection within KUPRI. We suggest this hypothesis because: (1) all of the rhesus and Japanese macaques used in this study were born in KUPRI, (2) *H*. *pylori* strains carrying the *cagA* genes that are highly related to Hp_TH2099 *cagA* may be spread in rhesus macaques housed in KUPRI, and (3) the rhesus macaques have ancestors that came to KUPRI more than 50 years ago from India.

Phylogenetic tree analysis revealed that the Hp_TH2099 *cagA* gene is most closely related to the *cagA* gene carried by the UM067 strain, which was isolated in Malaysia^35,46^. However, the Hp_TH2099 *cagA*-encoded CagA protein is quite unique in its amino acid sequence when compared to other ABC-type CagA proteins known to date. In particular, the EPIYA-repeat region of Hp_TH2099 CagA possesses hitherto unidentified variations in the EPIYA-C segment and the CM sequence that serve as binding sites for SHP2 and PAR1b, respectively. The structural modeling predicts that Gly substitutions of the highly conserved Arg residues in the first and second CM sequences of Hp_TH2099 CagA are responsible for the reduction in its PAR1b-binding activity, the observation that is consistent with the results of artificial mutation analysis for the CM sequence reported previously^38^. The Leu-to-Ile substitution in the first CM sequence of Hp_TH2099 CagA may additionally dampen interaction of Hp_TH2099 CagA with PAR1b.

The magnitude of hummingbird-phenotype induction by CagA is proportional to the level of the CagA-SHP2 complex formed in CagA-delivered cells^16,17^. CagA-SHP2 complex formation requires tyrosine phosphorylation of EPIYA-C and EPIYA-D in Western CagA and East Asian CagA, respectively^9^. Notably, the SHP2-binding affinity of CagA is determined by the residue located at the + 5 position from the tyrosine phosphorylation site of the EPIYA-C or EPIYA-D segment^9,13^. East Asian CagA, which contains Phe at pTyr + 5, exhibits ~100-fold greater SHP2-binding affinity than that of East Asian CagA, which possesses Asp at pTyr + 5^39^. In the EPIYA-C segment of Hp_TH2099 CagA, pTyr + 5 is Asn instead of highly conserved Asp in Western CagA, indicating that the alteration affects the ability of CagA to bind to SHP2. Indeed, Hp_TH2099-derived EPIYA-C binds to SHP2 more potently than canonical EPIYA-C does. Based on the crystal structure of the CagA-SHP2 interaction, Asp-to-Asn substitution at pTyr + 5 stabilizes the binding of EPIYA-C to the N-SH2 domain of SHP2 as Asn is less repulsive than Asp to the acidic surface of the N-SH2 cleft. On the other hand, we also find that the Hp_TH2099-derived EPIYA-C segment undergoes less tyrosine phosphorylation than does canonical EPIYA-C due to the hitherto unreported Pro-to-Leu substitution at pTyr-2 in the EPIYA-C segment. Notably, the Pro residue has been 100% conserved in EPIYA-C except for the case of the current study. Given that substrate specificity of tyrosine kinase is determined primarily by several residues immediately upstream of the phosphotyrosine^47^, the ELIYA sequence may be less well recognized than EPIYA by host tyrosine kinases. Collectively, the pTyr-2 substitution present in the EPIYA-C segment of Hp_TH2099 CagA might have greater negative effect on the tyrosine phosphorylation-dependent CagA activity compared to the positive effect that may be induced by the pTyr + 5 substitution. Through the compilation of multiple substitutions in the EPIYA-C segment as well as the CM sequence that give rise to the loss of PAR1b binding and the reduced tyrosine phosphorylation, the macaque-derived CagA exhibits substantially attenuated pathobiological activity compared to the canonical Western CagA.

*H*. *pylori* strains isolated from experimentally infected macaques frequently show loss of a functional TFSS by frameshift mutations in the *cagY* gene^22^. This is most probably due to a mutation burst that occurs during the acute phase of experimental *H*. *pylori* infection, which facilitates adaptation of *H*. *pylori* to the new host^48,49^. In contrast, *H*. *pylori* strains isolated from naturally infected macaques retain a functional TFSS that delivers CagA into the host cells^50^, indicating that natural *H*. *pylori* infection, which occurs at an early age through transmission from infected dams to newborns^51^, does not provoke strong inflammatory responses that trigger a mutation burst in the *H*. *pylori* genome. The macaque-derived Hp_TH2099 strain also retains a functional TFSS, supporting the idea that the strain has not undergone the mutation burst. Consistently, amino acid substitutions found in Hp_TH2099 CagA are not commonly associated with the mutation burst induced nu experimental *H*. *pylori* infection of macaques^47^.

Gastric organoids, either developed from ES cells or stomach tissues, have recently been applied to recapitulate *in vivo H*. *pylori* infection^28–31^. The present study revealed that injection of the *H*. *pylori* standard strain, NCTC11637 or 26695, into the lumen of ES-cell derived gastric organoids causes extensive destruction of the epithelial layer in a CagA-dependent manner. In this regard, CagA-PAR1b interaction has been shown to elicit junctional defects^15^. CagA also associates with E-cadherin^52^, possibly via PAR1b as an adaptor, and thereby perturbs E-cadherin function. In the *H*. *pylori*-injected organoids, disruption of the epithelial layer was associated with the loss of E-cadherin staining from the lateral membrane surface, suggesting that CagA-PAR1b-mediated malfunctioning of E-cadherin is involved in the epithelial damage. This notion was further supported by the finding that infection with the Hp_TH2099 strain, which delivers the CagA protein that cannot bind to PAR1b, was incapable of destroying the organoid epithelial layer. ES cell-derived organoids may therefore provide a useful tool in evaluating the relative strength of *cagA*-positive *H*. *pylori* virulence, which is primarily determined by the structural polymorphism of CagA^9,19^ as well as the efficiency of CagA delivery^53^.

Isolation of less pathogenic *H*. *pylori* strains carrying CagA with attenuated pathobiological activity in a small and isolated host population is reminiscent of the result of our previous study regarding v225d CagA, a natural CagA variant of *H*. *pylori* isolated from an Amerindian subject living in the Amazon rainforest^54^. Like Hp_TH2099 CagA, v225d CagA was also incapable of binding with PAR1b, suggesting that attenuated CagA activity, especially loss of PAR1b binding, is a hallmark of *H*. *pylori* sustainably cohabiting with a small number of hosts. Although it needs to be determined whether macaques carry and transmit *H*. *pylori* among themselves in wild life, many studies have shown that wild macaques have an intimate relationship with humans, especially in Asian countries^55,56^. It is possible that macaques became infected with *H*. *pylori* from human vomit or waste. Conversely, humans might have acquired *H*. *pylori* from macaques by feeding on them, a human habit that has been historically recorded in many geographic regions of the world. It is tempting to speculate that rapid evolution of distinct *H*. *pylori* subpopulations has been made possible by back and forth natural infection/colonization between humans and non-human primates, which may have facilitated bacterial microevolutions in a way that is milder than the mutation burst triggered by experimental *H*. *pylori* infection.

## Methods

### Macaques

Rhesus macaques (*Macaca mulatta*), a Taiwanese macaque (*Macaca cyclopsis*) and Japanese macaques (*Macaca fuscata*) housed at the Primate Research Institute, Kyoto University (KUPRI) were used in this study. DNAs were purified from stomach contents of macaques by using a DNeasy Blood & Tissue Kit (QIAGEN). Gastric juice was sampled through an oral or intranasal tube when the macaques were anesthetized (intramuscular administration of 5 mg/kg ketamine, 0.025 mg/kg medetomidine, and 0.125 mg/kg midazolam) or when physically restraint using squeezing cage for veterinary examinations. Twenty ml of gastric juice was obtained from each macaque, and the gastric juice was mixed with *Helicoporter*® (BML, Japan), stored at 4 °C, and 200 µl of the samples were plated onto a *Helicobacter* agar plate (Nissui) within 48 hours. Samples were incubated at 37 °C in 5% CO_2_ in an incubator until colonies grew.

## Amplification of the *H*. *pylori cagA* gene

PCR amplification of the *cagA* fragment from the gastric contents of macaques was conducted by using the forward primer <5′caagcaaaaagcgaccttg3′> and the reverse primer <5′gcaactatcttatcattcacgagctt3′>, which were designed to amplify an approximately 1,000-bp *cagA* fragment. Colony direct PCR was performed using the forward primer <5′aatacaccaacgcctccaag 3′> and the reverse primer <5′gttaccttgctgaaatccttatt3′>, which were designed to amplify an approximately 750-bp *cagA* fragment. PCR products were run on 0.8% agarose gel. Amplified DNA bands were cut, purified by using a Gene clean kit (Funakoshi) and cloned into pCR2.1 vector using a TA cloning kit (Sigma-Aldrich). Sequences were read by an ABI prism 3130 Genetic Analyzer (Applied Biosystems).

## Illumina whole-genome shotgun sequencing

The genome of the macaque-derived *H*. *pylori* Hp_TH2099 strain was sequenced on an Illumina MiSeq. Genomic DNA was extracted from *H*. *pylori* by using Nucleospin Microbial DNA (TAKARA) according to the manufacturer’s protocol. Samples were quantified using a Tape Station on a Genome chip (Agilent). Indexed adapters were from the NextERA Sample Prep Kit. Products were purified with Ampure XT (Beckman Coulter). The purified samples were then enriched by eight PCR cycles according to the manufacturer’s protocol. An aliquot of the library was run on the Tape Station on a Genome chip and the library fragments were found to be approximately 600 bps on average. Sample concentrations were quantified using Qubit. DNA was loaded onto the MiSeq following the protocol for Illumina MiSeq Reagent Nano kit v2 (300 cycles). Sequencing was performed as a 150 × 150 bp paired-end run with overlapping reads to an average 200 coverage.

### Sequence and bioinformatic analysis

Sequence analysis of full-length *cagA* genes was performed by MUSCLE and Clustal W. Reference *cagA* genes (199 genes) were collected from NCBI database (http://www.ncbi.nlm.nih.gov). Identical sequences and sequences that are not reported as a CagA protein were removed and phylogenetic relationships were further analyzed by the neighbor-joining method with 1000 bootstrap steps.

### MLST analysis

For multi-locus sequence typing (MLST) of the *H*. *pylori* Hp_TH2099 genome, fragments of seven unlinked housekeeping genes (*atpA*, *efp*, *ureI*, *ppa*, *mutY*, *trpC*, and *yphC*) were extracted by using BLAST. A set of 430 sequences used in previous studies was retrieved from an MLST database (http://pubmlst.org/helicobacter) and aligned and then phylogenetic trees were drawn by using mafft (http://mafft.cbrc.jp/alignment/Software/).

### fineSTRUCTURE analysis

Population assignment of the *H*. *pylori* Hp_TH2099 strain was conducted by means of inference of a global population structure at a fine scale from genome-wide haplotype data. In addition to the genome sequence of the Hp_TH2099 strain, genome sequences of 199 strains of hspAfrica1SAfrica, hspAfrica1WAfrica, hpAfrica2, hpAsia2, hspEuropeN, hpEastAsia, and hspAmerind that were analyzed recently were used^34^. Pairwise genome alignment was first conducted between the reference 26695 strain and one of the 199 strains by progressiveMauve^57^, which can construct positional homology alignments even for genomes with variable gene content and rearrangement. The obtained alignments were then combined into a multiple whole-genome alignment, in which each position corresponded to that of the reference genome. SNP calling was conducted, while preserving information of SNP positions, to prepare genome-wide haplotype data. Imputation of polymorphic sites with a missing frequency <2% was conducted using BEAGLE software^58^. Chromosome painting and fineSTRUCTURE were then used according to a procedure for applying them to *H*. *pylori* genomes as described previously^39,59,60^. Briefly, ChromoPainter (version 0.04) was used to infer chunks of DNA donated from a donor to a recipient for each recipient haplotype, and the results were summarized into a “co-ancestry matrix” that contains the number of recombination-derived chunks from each donor to each recipient individual. Then fineSTRUCTURE (version 0.02) for 100,000 iterations of both the burn-in and Markov chain Monte Carlo (MCMC) chain was ran in order to conduct clustering of individuals based on the co-ancestry matrix.

### Construction of expression vectors

The pSRα-based mammalian expression vector for C-terminal FLAG-tagged ABC’ (ABC’-CagA-FLAG) was made using a synthesized *cagA* gene (*cagA*^*Hs*^), which encodes the NCTC11637 standard strain-derived ABCCC type CagA. In the *cagA*^Hs^ gene, codon usage of the bacterial *cagA* gene was optimized for mammalian expression^20^. After adding the nucleotide sequence encoding the C-terminal FLAG-tag, the *cagA*^Hs^ gene was inserted into the pSP65SRα mammalian expression vector to make pSRα-ABCCC-CagA^Hs^-FLAG. To make an expression vector for ABC’-CagA-FLAG (pSRα-ABC’-CagA-FLAG), the humanized *cagA* region encoding triple EPIYA-C segments and the second (distal) CM sequence in pSRα-ABCCC-CagA^Hs^-FLAG was replaced by the corresponding nucleotide sequence in the Hp_TH2099 *cagA* gene. An ABC-CagA-FLAG vector (pSRα-ABC-CagA-FLAG) was also made from pSRα-ABCCC-CagA^Hs^-FLAG by internal deletion of the *cagA* gene sequence encoding the first and second EPIYA-C segments for control experiment.

### Cell culture and transfection

AGS human gastric epithelial cells were cultured in RPMI 1640 medium supplemented with 10% fetal bovine serum (FBS) (GIBCO). COS7 cells, which are derived from African Green Monkey kidney, were cultured in DMEM medium supplemented with 10% FBS. Expression vectors were transiently transfected into cells using Lipofectamine 2000 reagent (Invitrogen) according to the manufacturer’s instructions.

### Immunoprecipitation and immunoblotting

Immunoprecipitation and immunoblotting were performed as described previously^15,16^.

### Cell morphological analysis

AGS cells were transiently transfected with CagA expression vectors. Morphology of the cells was observed 24 hours after transfection. Fluorescent microscopic observation and statistics were performed as described previously^17,41^.

### Antibodies

The antibodies used in this study are listed below with the target, species, company, catalogue number, and dilution. The primary antibodies for immunoprecipitation and immunoblotting are: Actin, goat, Santa Cruz, sc1615, 1:1,000, SHP2, rabbit, Santa Cruz, sc280, 1:1,000; PAR1b, rabbit, abcam, EPR 8553, 1:1,000; FLAG, mouse, Sigma, F3165, 1:1000; phosphotyrosine, mouse, Millipore, 05–1050, 1:1000. The primary antibodies used for immunostaining are: α-SMA, mouse, Sigma, A5228, 1:100; β-Catenin, rabbit, SantaCruz, sc7199, 1:100; CagA, rabbit, AUSTRAL Biologicals, HPP5003-9, 1:100; E-cadherin, mouse, BD Biosciences, 610182, 1:100; E-cadherin, goat, R&D Systems, AF648, 1:100; Gastrin, mouse, Santa Cruz, sc28302, 1:100; *H*. *pylori*, rabbit, Abcam, ab80519, 1:100; Muc5AC, mouse, Abcam, ab3649, 1:100; Muc6, mouse, Abcam, Ab49462, 1:100; Nanog, rabbit, Abcam, ab21624, 1:500; Oct3/4, mouse, Santa Cruz, sc5279, 1:500; Vimentin, goat, Santa Cruz, sc7557, 1:200; ZO-1, rabbit, Invitrogen, 617300, 1:100.

### *H*. *pylori* infection

Three *H*. *pylori* Western standard strain NCTC11637, 26695, G27, an isogenic strain lacking *cagA* (NCTC11637*∆cagA*), an isogenic strain lacking *cag*PAI (G27*∆cag*PAI) and the Hp_TH2099 strain were grown on blood agar plates consisting of Trypticase Soy agar (BD) with 5% horse blood (Colorado Serum Company). For infection to AGS cells, *H*. *pylori* were resuspended in RPMI 1640 medium and incubated with cells for 5 hours, at a MOI of 200. After incubation, cells were harvested and lysed with 0.1% saponin for 10 min, to eliminate the protein extraction from *H*.*pylori*. For organoid injections, *H*. *pylori* were resuspended in Brucella Broth at a concentration of 1 × 10^8^ bacteria/ml and loaded onto the Nanoject II (Drummond) microinjector apparatus. One hundred nl (containing 1 × 10^4^ bacteria) was injected directly into the lumen of each organoid that were about 1 mm in diamter, and injected organoids were cultured for an additional 12 hours. Brucella Broth was injected as a negative control. For all experiments, antibiotics were removed from the medium 24 hours before *H*. *pylori* infection.

### Equipment and settings

For all blots, chemiluminescent signals were detected and recorded by either LAS 4000 (Fuji Film) bioimage analyzer or exposure of the membranes to Amersham Hyperfilm^TM^ ECL (GE Healthcare). Pictures were cropped with Adobe Photoshop CC 2018 (Adobe systems incorporated). Microscopic images were obtained using FLUOVIEW-FV1200 (Olympus) confocal microscope systems.

### Ethical approval

This study was conducted under the Guide for Care and Use of Laboratory Primates of the Primate Research Institute, Kyoto University (KUPRI) and approved by the Animal Care and Use Committee of Kyoto University (protocol number: 2017-76).

## Electronic supplementary material

Supplementary Information

## Data Availability

For Hp_TH2099, the genome sequence reads obtained are available from the National Center for Biotechnology Information (NCBI) Genebank under the accession number CP025748. The entire sequences of the *cagA* genes amplified from the stomach contents of rhesus macaques Mm1874 and Mm1887 have been submitted to the NCBI GenBank under the accession number MG981049 and MG981050. The BioProject accession number of the study is PRJNA429063. The BioSample accession number is SAMN08328770.
