# Supplementary material for: Evaluating the origin and virulence of a Helicobacter pylori cagA-positive strain isolated from a non-human primate
Source: Sci Rep. 2018 Oct 29;8:15981. doi: 10.1038/s41598-018-34425-4 (PMC6206097; doi:10.1038/s41598-018-34425-4)
Supplement: Supplementary file 1 — Supplementary Information [file 41598_2018_34425_MOESM1_ESM.pdf]

Supplementary Information

Evaluating the origin and virulence of a *Helicobacter pylori* *cagA*-positive strain isolated from a non-human primate

Kana Hashi, Chihiro Imai, Koji Yahara, Kamrunnesa Tahmina, Takeru Hayashi, Takeshi Azuma, Takako Miyabe-Nishiwaki, Hideyuki Sato, Masao Matsuoka, Sachi Niimi, Munehiro Okamoto and Masanori Hatakeyama

Supplementary methods

RNA isolation and qPCR. Total RNA was isolated from *H. pylori*-infected AGS cells and COS7 cells using Trizol reagent (Invitrogen). Reverse transcription was performed with 4 μg RNA using SuperScript™ II Reverse Transcriptase (Invitrogen) according to the manufacturer's protocol. The qPCR was performed using SYBR® *Premix Ex Taq*™ II (Tli RNaseH plus) (Takara) on a StepOnePlus Real-Time PCR System (Applied Biosystems). Analysis was performed using the ΔΔCt method. PCR primers were designed using Primer 3 plus (http://www.bioinformatics.nl/cgi-bin/primer3plus/primer3plus.cgi). Following primers were used; AGS IL-8, 5'cctgatttctgcagctctgtgtgaag 3' and 5'attgcatctggcaaccctac3'; AGS GAPDH, 5'tgaaggtcggagtcaacggatttggt3' and 5'catgtgggccatgaggtccaccac3'. COS7 IL-8, 5'agaactgagagtgattgagagtgg3' and 5'ctcagccctcttcacaaacttc3'; COS7 GAPDH, 5'atcatctctgccccctctgctgac3' and 5'agtgagcttcccgttcagctc3'.

Construction of the Hp_TH2099 Δ*cagA* isogenic strain. The Hp_TH2099 *cagA* gene was amplified from the Hp_TH2099 genome by polymerase chain reaction (PCR) using specific primers and then cloned into in the pBlueScript II SK(+) vector. A targeting vector of the Hp_TH2099 *cagA* gene was constructed by insertion of a kanamycin-resistance gene cassette derived from the pENTR3c plasmid into the Hp_TH2099 *cagA* gene according to a standard protocol[1]. The resulting targeting vector (50 μg) was added to 2 ml of the Hp_TH2099

strain suspension (5 x 10^8 /ml) in Brucella Broth medium. After incubation for 12 h, bacteria were plated and grown on blood agar plates consisting of Trypticase Soy agar (BD) with 5% horse blood (Colorado Serum Company) containing 30 μg/ml Kanamycin to select for the kanamycin-resistant transformant obtained after 5 days according to a standard procedure[1]. The grown colony was isolated and further expanded on plates. The *cagA* gene disruption and the lack of CagA expression were confirmed by sequencing, PCR analysis and an anti-CagA immunoblotting.

Differentiation of ES-cell derived organoids to definitive endoderm. The mouse ES cell line EB3 (derived from 129/Ola strain, 9 passages) was kindly provided by Dr. Hitoshi Niwa (Center for Developmental Biology, RIKEN, Japan) and was cultured in Glasgow's MEM (GMEM) containing 10% FBS, 0.1 mM non-essential amino acids (NEAA; Sigma-Aldrich), 0.1 mM β-mercaptoethanol (Nacalai Tesque), and 1x penicillin/streptomycin (Wako) supplemented with 1 unit/ml mouse recombinant leukemia inhibitory factor (LIF) (Nacalai Tesque), 3 μM CHIR99021, 2 μM Y27632, and 1 μM PD0325901 at 37℃ in 5% CO2. There was no sign of mycoplasma contamination by a test using a Micoalart Kit (Lonza). For EB formation, mouse ES cell colonies were dissociated completely into single cells with a 0.25% trypsin–EDTA solution (Wako). Floating ES cells were collected and seeded at 2.5 x 10^5 cells on a low-attachment 10 cm petri dish in EB medium [IMDM

containing 10% FBS, 200 μg/ml transferrin, 0.45 mM 1-thioglycerol, 50 μg/ml ascorbic acid, 2 mM l-glutamine and 1x penicillin/streptomycin (Wako)]. For differentiation, EBs were accumulated in a 15 ml falcon tube per dish and were re-suspended and plated in a Matrigel-coated 24-well dish using 0.25% Trypsin-EDTA. On the next day, stem cells were differentiated to the definitive endoderm.

Supplementary Figures

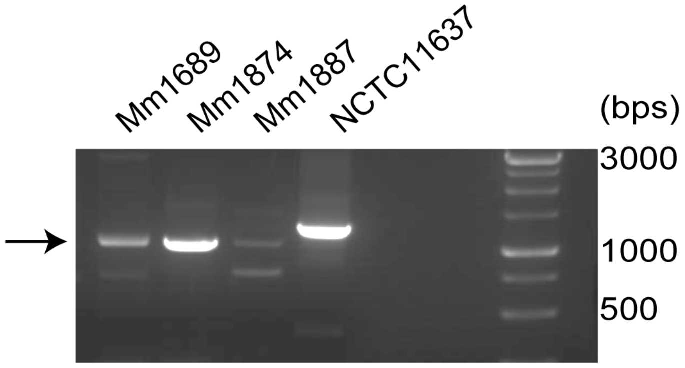

Supplementary Figure S1. Amplification of *cagA* fragment from gastric juices of rhesus macaques. The *cagA* fragment was amplified by PCR from DNA samples derived from gastric juice of rhesus macaques and run by using agarose gel. Mm1689, Mm1874 and Mm1887 are DNA samples obtained from each of the macaques. "NCTC11637" indicates DNA isolated from the *H. pylori* standard strain NCTC11637, which carries the ABCCC-type Western CagA, as a positive control. The arrow indicates the position of ~1000-bp fragment, which is specifically amplified by the *cagA* primers. M; 1kb-ladder marker.

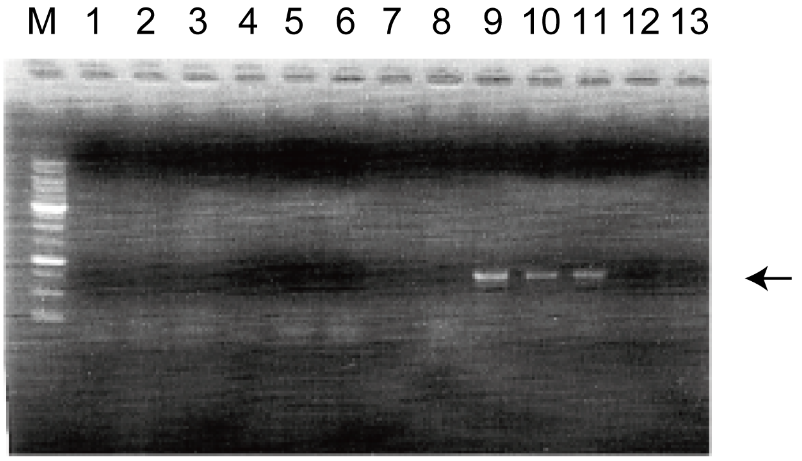

Supplementary Figure S2. Colony directed PCR of the *H. pylori cagA* gene. A *cagA* gene fragment was specifically amplified by PCR from bacterial colonies that were isolated from gastric juices of a Japanese macaque TH2099. The arrow indicates the position of the amplified *cagA* fragment (762-bp). M; 1kb ladder marker. Thirteen bacterial colonies (numbered from 1 to 13) were randomly picked up and subjected to the PCR analysis.

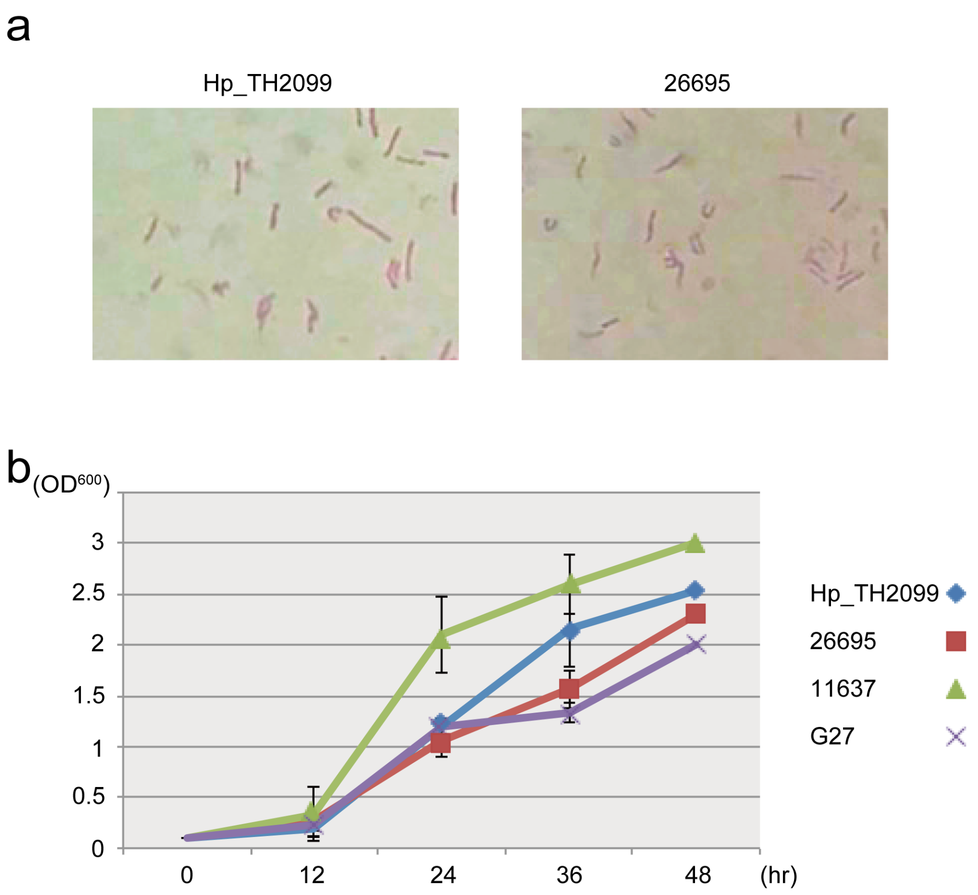

c

Biological Test	Results on Hp_TH2099
Urease	Positive
Oxidase	Positive
Catalase	Positive

Supplementary Figure S3. Investigation of the characteristics of Hp_TH2099. (a) Gram staining of Hp_TH2099 and 26695 strains. **(b)** Growth curve of Hp_TH2099, 26695, 11637 and G27 *H. pylori* strains in Brucella Broth with 10% FBS. OD: optical density. **(c)** Results of biological tests of Hp_TH2099. Urease activity was measured by EB-20 (Nissui). Oxidase activity was measured by Oxidase Swabzyme (EY Laboratories) and catalase activity was measured by adding 3% H_2O_2 to the Hp_TH2099 culture.

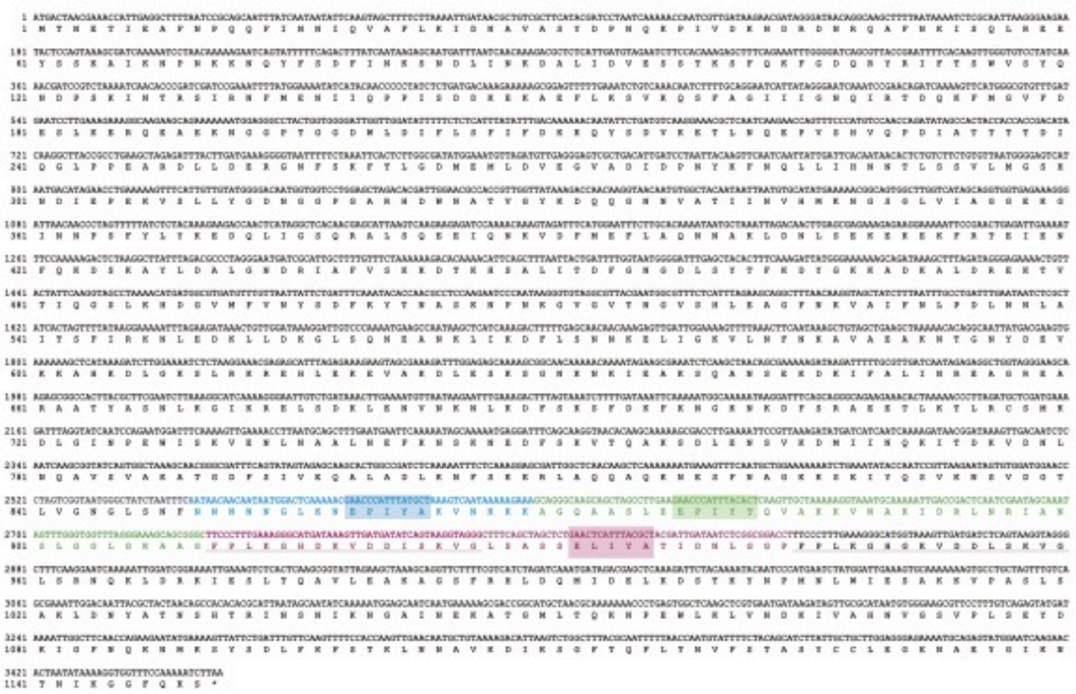

Supplementary Figure S4. **Nucleotide sequences of Hp_TH2099** ***cagA*** **gene.** The full-length sequence of the Hp_TH2099 *cagA* gene and its product were extracted from whole genome sequences and translated. Blue, EPIYA-A segment; Green, EPIYA-B segment; Purple, EPIYA-C segment. Colored boxes indicate the EPIYA motifs and underlines indicate CM sequences.

a

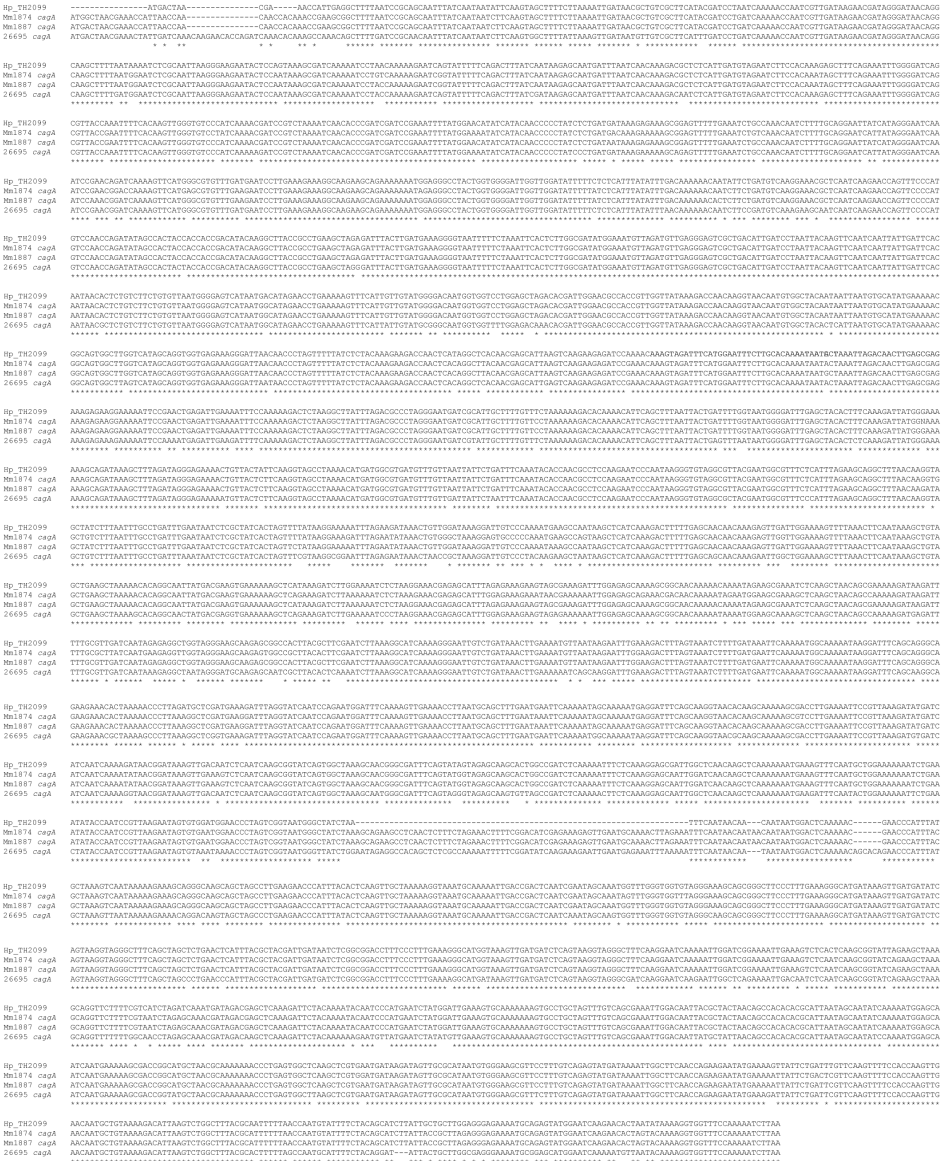

Identity (%)				
	Hp_TH2099	Mm1874 *cagA*	Mm1887 *cagA*	26695 *cagA*
Hp_TH2099		96	97	92
Mm1874 *cagA*			98	92
Mm1887 *cagA*				92
26695 *cagA*				

b

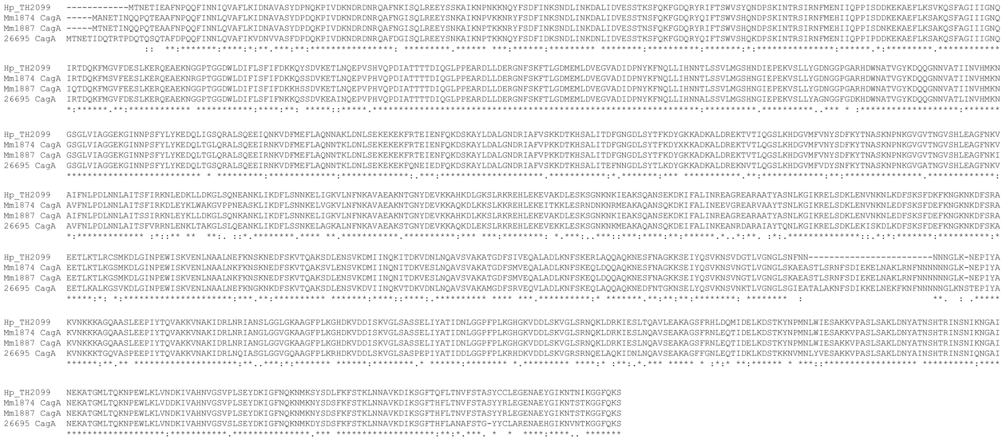

Identity (%)				
	Hp_TH2099	Mm1874 CagA	Mm1887 CagA	26695 CagA
Hp_TH2099		92	94	86
Mm1874 CagA			96	86
Mm1887 CagA				86
26695 CagA				

c

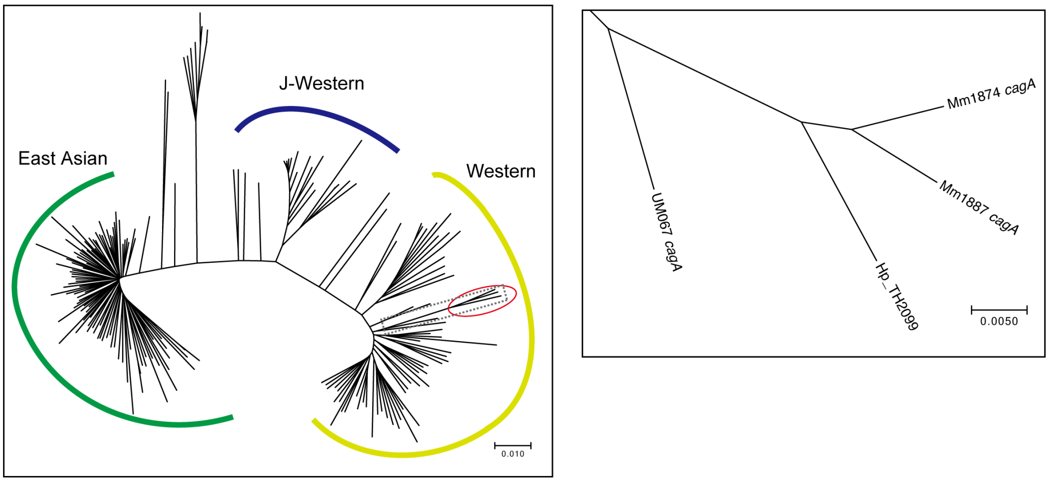

d

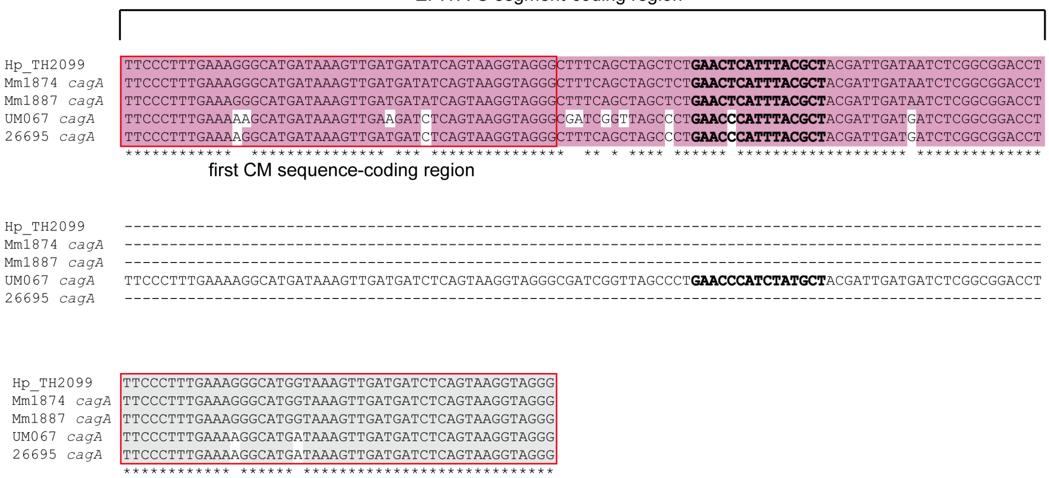

Identity (%)					
	Hp_TH2099	Mm1874 *cagA*	Mm1887 *cagA*	UM067 *cagA*	26695 *cagA*
Hp_TH2099		100	100	91	95
Mm1874 *cagA*			100	91	95
Mm1887 *cagA*				91	95
UM067 *cagA*					96
26695 *cagA*					

e

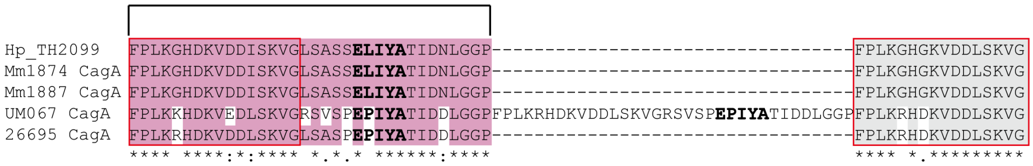

Identity (%)					
	Hp_TH2099	Mm1874 CagA	Mm1887 CagA	UM067 CagA	26695 CagA
Hp_TH2099		100	100	82	86
Mm1874 CagA			100	82	86
Mm1887 CagA				82	86
UM067 CagA					96
26695 CagA					

Supplementary Figure S5. Comparison of macaque-isolated *cagA*/CagA with canonical ABC-type Western *cagA*/CagA. Pair-wise alignments were carried out by Clustal W software. **(a)** Alignment of full-length nucleotide sequences between Hp_TH2099, Mm1874, Mm1887, and 26695 *cagA* genes. **(b)** Alignment of full-length amino acids sequences between Hp_TH2099, Mm1874, Mm1887, and 26695 CagA proteins. **(c)** A phylogenetic tree was constructed by adding full-length sequences of Hp_TH2099, Mm1874, Mm1887 *cagA* genes to the 199 reference *cagA* genes used in Figure 2c. Red circle indicated the positions of Hp_TH2099, Mm1874, and Mm1887 *cagA* genes (left). Magnified view of the black dotted box in the phylogenetic tree (right). **(d)** Alignment of nucleotide sequences of Hp_TH2099, Mm1874, Mm1887, UM067, and 26695 *cagA* gene segments encoding the EPIYA-C segment and the second (distal) CM sequence. Notably, the UM067 *cagA* gene contains tandem duplication of the nucleotide sequence encoding the EPIYA-C segment. Identical sequences are highlighted in purple. Nucleotide identities (%) are shown in the table. **(e)** Alignment of amino acid sequences of Hp_TH2099, Mm1874, Mm1887, UM067, and 26695 CagA protein regions that correspond to the EPIYA-C segment and the second (distal) CM sequence. The UM067 CagA protein is an ABCC-type Western CagA containing two identical EPIYA-C segments in tandem. Identical amino acid sequences are highlighted in purple. Amino acid identities (%) are shown in the table.

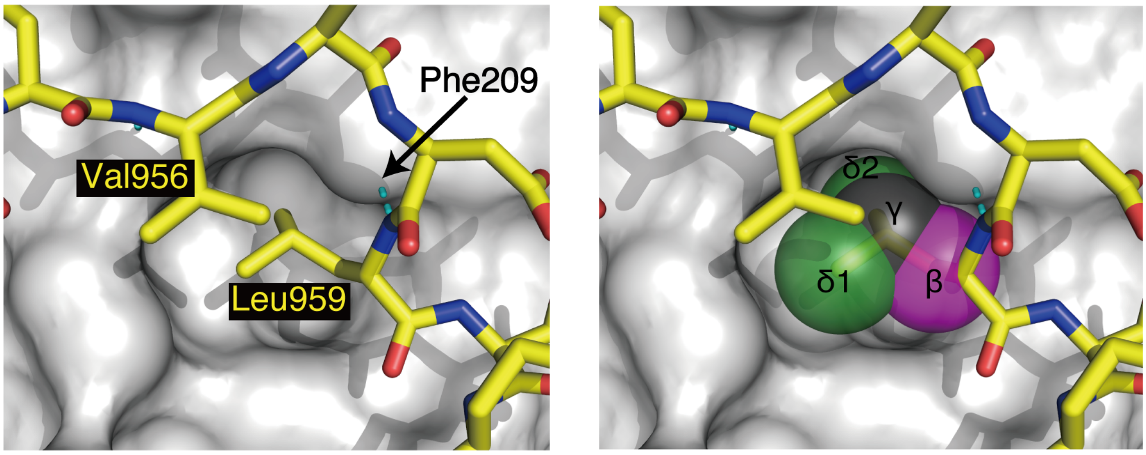

Supplementary Figure S6. Role of the Leu residue at the 12th position from the N-terminus of the canonical CM sequence in binding of CagA with PAR1b. Structural basis for the interaction of PAR1b (grey) with Leu959 *H. pylori* 26695 CagA, which is located at the 12th position from the N-terminus in the second CM sequence (yellow) The binding interface was created using the data of the previous work reported by Nesić and co-workers (*Nat. Struct. Mol. Biol.* **17**, 130–132, 2010, PDB ID: 3IEC) (left). Carbons in the side chain of Leu959 were represented by ball models with distinct colors; β-, γ- and δ1/2-carbons are shown in purple, dark grey and green, respectively (right). Main chain of Leu959 was fixed by the hydrogen bond formation (cyan dashed line) with Phe209 residue in PAR1b. The sequence corresponding to Leu959 in 26695 CagA is replaced by Ile (Ile922) in Hp_TH2099 CagA.

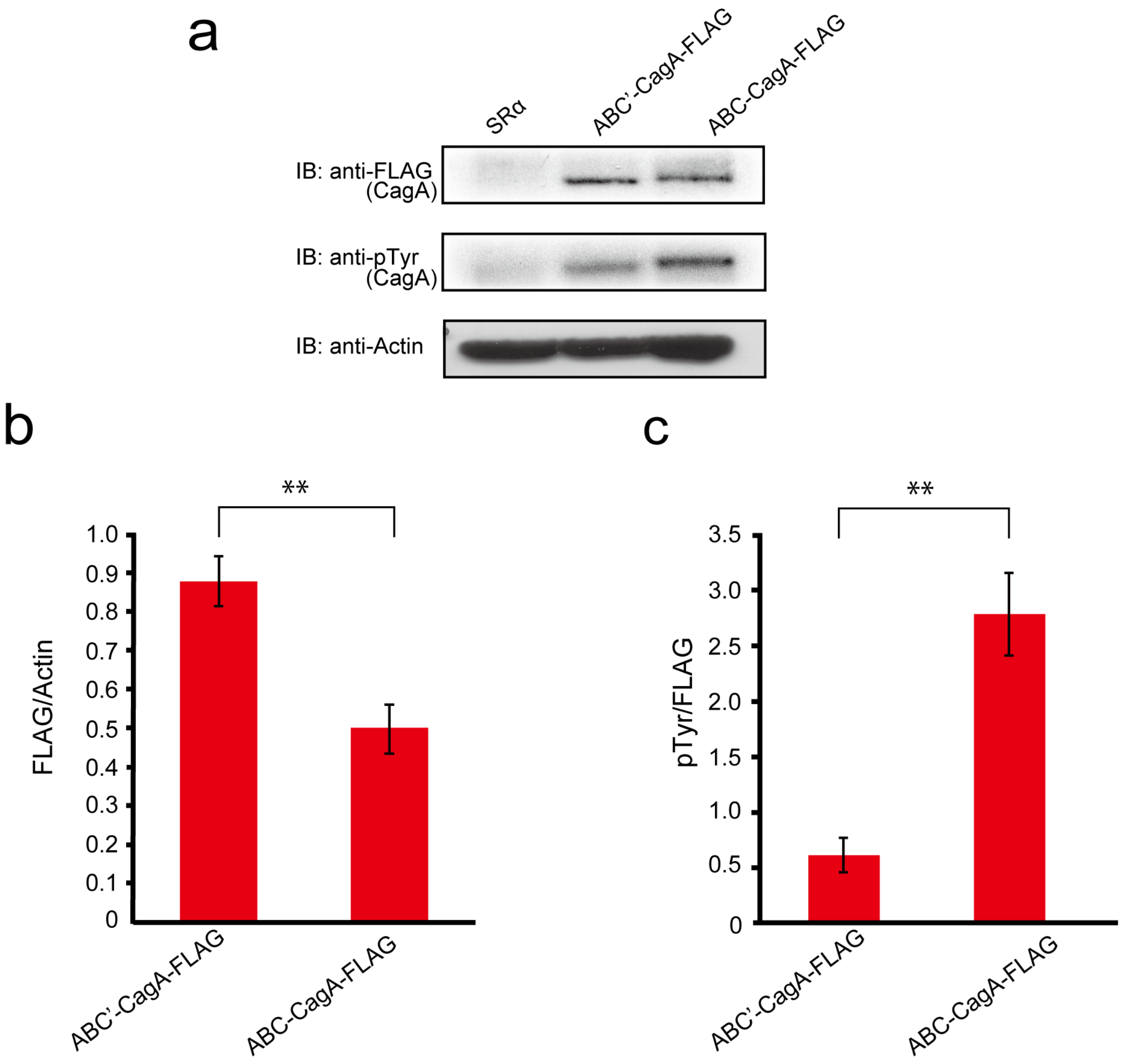

Supplementary Figure S7. Expression and tyrosine phosphorylation of transfected CagA in AGS cells. (a) Immunoblot analysis of transfected ABC'-CagA-FLAG and ABC-CagA-FLAG in AGS cells used for the hummingbird assay in Fig. 4d. Full-length blots are presented in Supplementary Figure S12. **(b, c)** Intensities of each protein bands were quantitated by ImageJ software. The FLAG/Actin ratios **(b)** and the phosphotyrosine (pTyr)/FLAG ratios **(c)** were then calculated and statistically analyzed by using Student's *t*-test. Error bars represent ± S.D. (n = 3), **: $p < 0.01$.

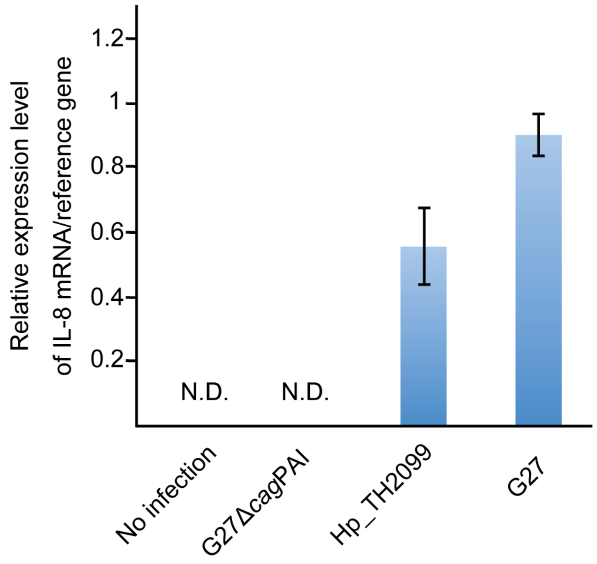

Supplementary Figure S8. IL-8 mRNA expression analysis in Hp_TH2099 infected COS7 cells. Hp_TH2099, G27Δ*cag*PAI and G27 strains were infected with African green monkey derived COS7 cells. RNAs were extracted from COS7 cells after 5 hours of infection and quantitative RT-PCR analysis was performed for measuring IL-8 mRNA expression levels. G27 is the *H. pylori* standard strain and G27Δ*cag*PAI is an isogenic strain of G27 that lacks the functional *cag*PAI. N.D.: not detected.

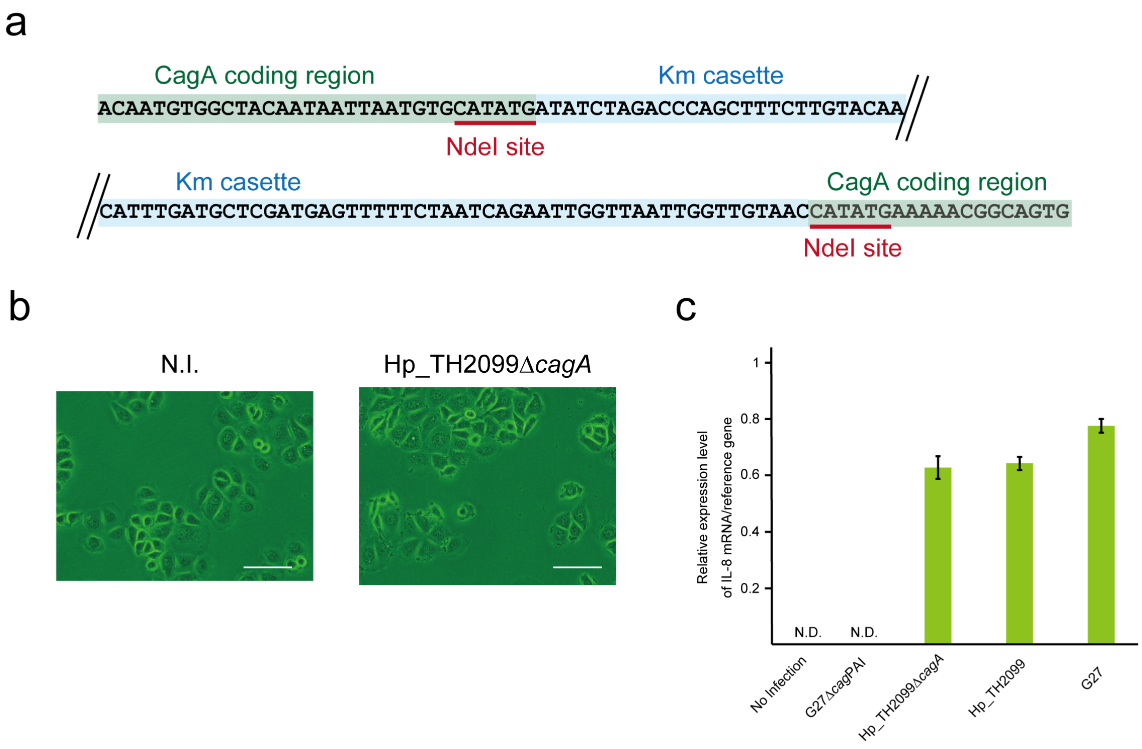

Supplementary Figure S9. Generation and characterization of Hp_TH2099Δ*cagA*. **(a)** The *cagA* nucleotide sequence of Hp_TH2099Δ*cagA* was read to confirm the disruption of the *cagA* coding region. **(b)** AGS cells were infected with the *H. pylori* Hp_TH2099Δ*cagA* for 24 hours at MOI of 300. Scale bars: 100 μm. **(c)** IL-8 mRNA expression in AGS cells. AGS cells were infected with the Hp_TH2099Δ*cagA*, parental Hp_TH2099, G27Δ*cag*PAI, or parental G27 strain. RNAs were extracted from AGS cells at 5 hours after the onset of *H. pylori* infection and were subjected to quantitative RT-PCR analysis for the expression of IL-8 mRNA.

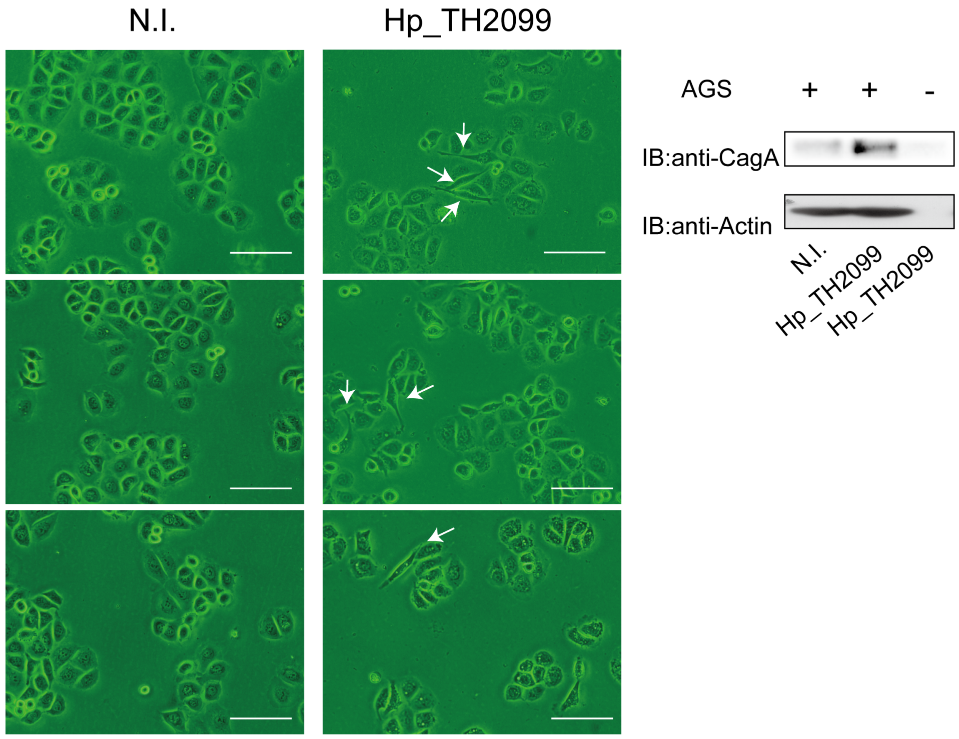

Supplementary Figure S10. Induction of the hummingbird phenotype by infection with the *H. pylori* TH2099 strain. AGS cells were infected with the *H. pylori* Hp_TH2099 for 24 hours at MOI of 300. Microscopic views of AGS cells with or without Hp_TH2099 infection. White arrows indicate cells showing the hummingbird phenotype. Scale bars: 100 µm (left). Saponin (0.1%) extracts of non-infected (N.I.) or Hp_TH2099-infected AGS cells were immunoblotted with an anti-CagA or an anti-Actin antibody. The Hp_TH2099 strain cultured without AGS cells in RPMI medium were also collected and lysed by 0.1% saponin as a negative control. Full-length blots are presented in Supplementary Figure S12.

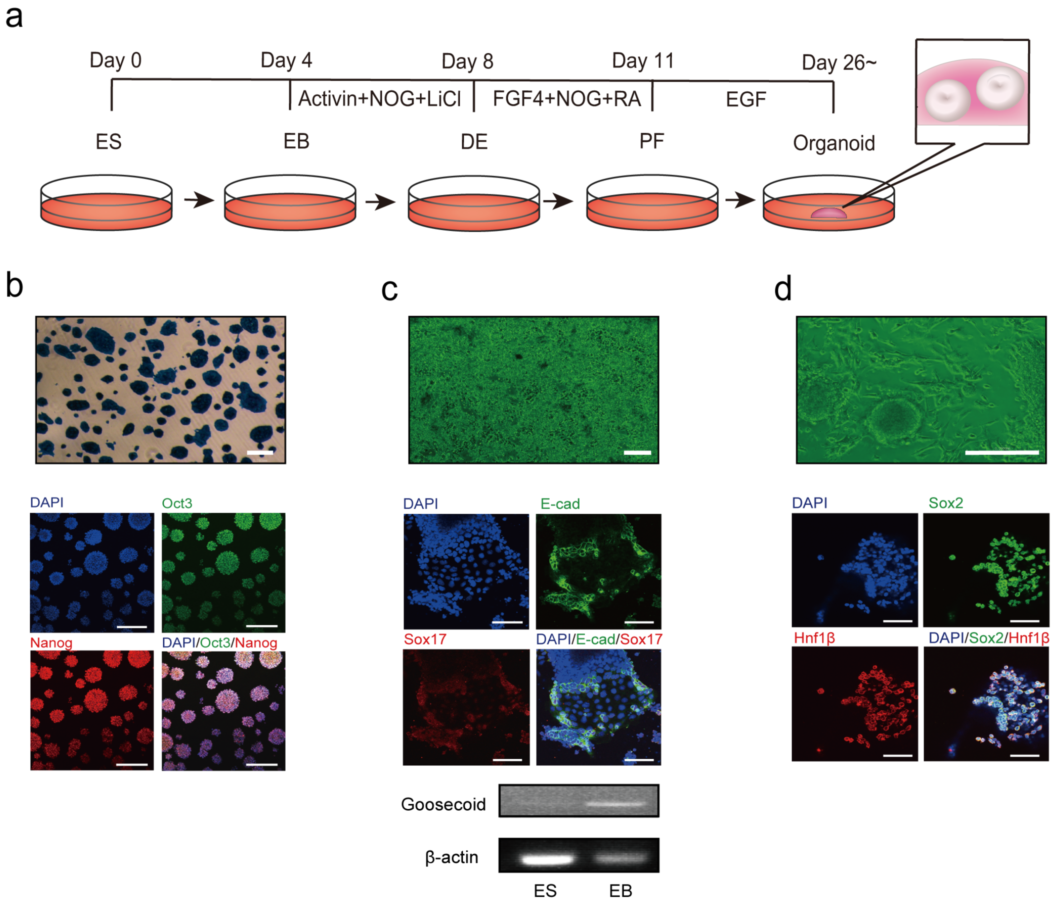
a
Day 0
Day 4
Day 8
Day 11
Day 26~
Activin+NOG+LiCl
FGF4+NOG+RA
EGF
ES
EB
DE
PF
Organoid
b
DAPI
Oct3
Nanog
DAPI/Oct3/Nanog
c
DAPI
E-cad
Sox17
DAPI/E-cad/Sox17
Goosecoid
β-actin
ES
EB
d
DAPI
Sox2
Hnf1β
DAPI/Sox2/Hnf1β

Supplementary Figure S11. Identification of ES cells, definitive endoderm, and posterior foregut. (**a**) A schematic view of the generation of organoids from mouse embryonic stem (ES) cells. Time line of the induction of organoids is shown. EB, embryonic body; DE, definitive endoderm; PF, posterior foregut, NOG: noggin, FGF4: mouse fibroblast growth factor 4, RA: retinoic acid, EGF: mouse epidermal growth factors. (**b**) Alkaline phosphatase assay of mouse ES cells. Scale bar; 200 μm (upper). Immunostaining analysis for the expression of embryonic stem cell markers, Oct3 and Nanog, in mouse ES cells. Scale bar; 50 μm (lower). (**c**) A microscopic image of definitive endoderm. Scale bar; 200 μm (upper). Immunostaining analysis for the expression of definitive endoderm-specific markers, E-cadherin and Sox17. Scale bar; 50 μm (middle). RT-PCR analysis of Goosecoid expression. ES, embryonic stem cells; DE, definitive endoderm (lower). (**d**) A microscopic image of the ES-derived spheroids at day 11. Scale bar; 200 μm (upper). Immunostaining analysis for the expression of the posterior foregut markers, Sox2 and Hnf1β, in the spheroids at day 11. Scale bar; 50 μm (lower).

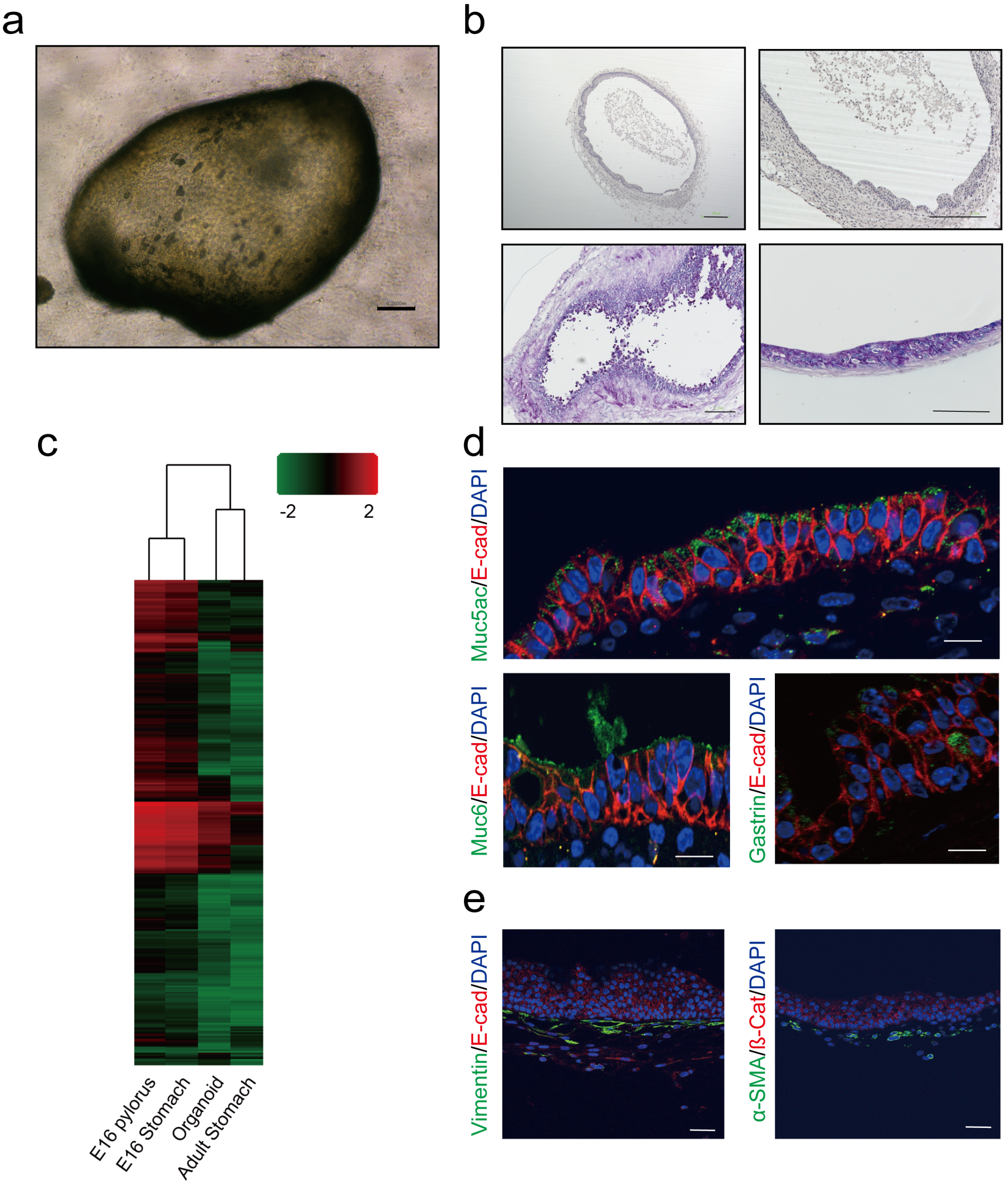
a
b
c
d
e
-2
2
E16 pylorus
E16 Stomach
Organoid
Adult Stomach
Muc5ac/E-cad/DAPI
Muc6/E-cad/DAPI
Gastrin/E-cad/DAPI
Vimentin/E-cad/DAPI
α-SMA/ß-Cat/DAPI

Supplementary Figure S12. Generation of gastric organoids from mouse ES cells. (**a**) A microscopic image of an ES cell-derived organoid at day 40. Scale bars: 200 μm. (**b**) HE staining (upper) and Alcian blue-PAS staining (lower) of an organoid at day 40. All scale bars: 200 μm. (**c**) A heat map representation of microarray data obtained using RNAs prepared from the ES-cell derived organoids at day 26, two fetal mouse stomach samples and an adult mouse stomach sample. E16 pylorus: RNA extracted from the pyloric region of the stomach of embryonic day 16 mouse. E16 stomach: RNA extracted from the stomach of embryonic day 16 mouse. Adult stomach: RNA extracted from the adult mouse stomach. (**d, e**) Immunostaining analysis of ES-cell derived organoids at day 40. Muc5ac, Mucin 5ac; Muc6, Mucin 6; E-cad, E-cadherin; β-cat, β-catenin; α−SMA, α-smooth muscle actin. Scale bars: 10 μm for e, 50 μm for f.

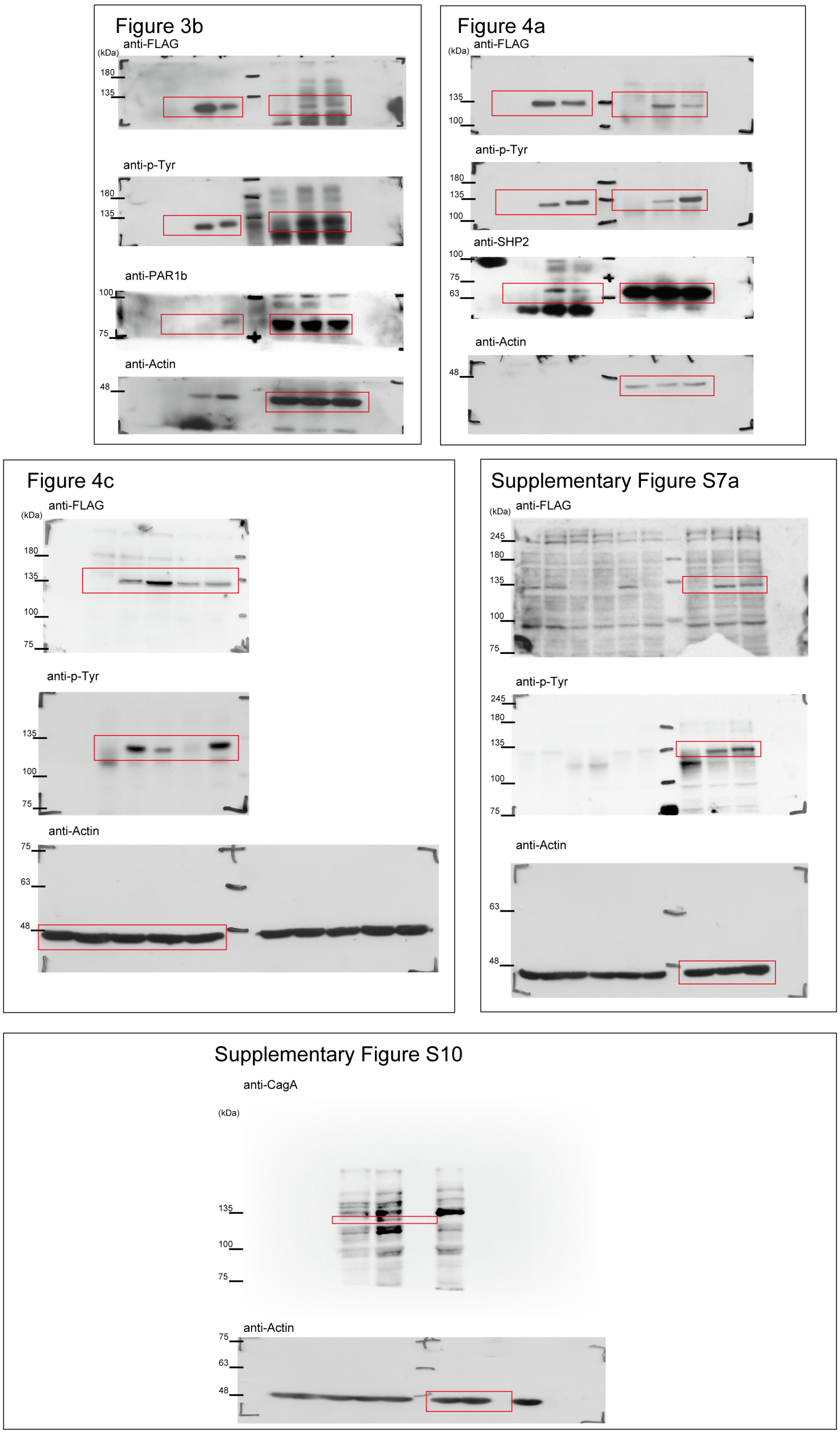

Supplementary Figure S13. Full length blots for Figure 3b, 4a, 4c, Supplementary Figure S7a and S10. Full scans of western blotting panels. Red squares indicate where the images were cropped.

Supplementary Table S1. Detailed information of isolates used for population assignment, ordered based on fineSTRUCTURE.

Isolate ID	Geographical source	Country	City/region	Assigned population	Reference
SA162A	South Africa	Mpumalanga	Ogies, Mpumalanga	hspAfrica1SAfrica	Didelot et al. 2013
SA227A	South Africa	Mpumalanga	Ogies, Mpumalanga	hspAfrica1SAfrica	Didelot et al. 2013
SA301A	South Africa	Mpumalanga	Ogies, Mpumalanga	hspAfrica1SAfrica	Didelot et al. 2013
SA220A	South Africa	Mpumalanga	Ogies, Mpumalanga	hspAfrica1SAfrica	Didelot et al. 2013
SA161A	South Africa	Mpumalanga	Ogies, Mpumalanga	hspAfrica1SAfrica	Didelot et al. 2013
SA214A	South Africa	Mpumalanga	Ogies, Mpumalanga	hspAfrica1SAfrica	Didelot et al. 2013
SA156A	South Africa	Mpumalanga	Ogies, Mpumalanga	hspAfrica1SAfrica	Didelot et al. 2013
SA226A	South Africa	Mpumalanga	Ogies, Mpumalanga	hspAfrica1SAfrica	Didelot et al. 2013
SA168A	South Africa	Mpumalanga	Ogies, Mpumalanga	hspAfrica1SAfrica	Didelot et al. 2013
SA146A	South Africa	Mpumalanga	Ogies, Mpumalanga	hspAfrica1SAfrica	Didelot et al. 2013
SA46C	South Africa	Mpumalanga	Ogies, Mpumalanga	hspAfrica1SAfrica	Didelot et al. 2013
SA30A	South Africa	Mpumalanga	Ogies, Mpumalanga	hspAfrica1SAfrica	Didelot et al. 2013
SA158A	South Africa	Mpumalanga	Ogies, Mpumalanga	hspAfrica1SAfrica	Didelot et al. 2013
SA35A	South Africa	Mpumalanga	Ogies, Mpumalanga	hspAfrica1SAfrica	Didelot et al. 2013
SA45A	South Africa	Mpumalanga	Ogies, Mpumalanga	hspAfrica1SAfrica	Didelot et al. 2013
SA170C	South Africa	Mpumalanga	Ogies, Mpumalanga	hspAfrica1SAfrica	Didelot et al. 2013
SA157A	South Africa	Mpumalanga	Ogies, Mpumalanga	hspAfrica1SAfrica	Didelot et al. 2013
Gambia94/24	West Africa	Gambia	n/a	hspAfrica1WAfrica	Human Microbiome Project
GAM260Bi	West Africa	Gambia	n/a	hspAfrica1WAfrica	Human Microbiome Project
GAM120Ai	West Africa	Gambia	n/a	hspAfrica1WAfrica	Human Microbiome Project
GAM118Bi	West Africa	Gambia	n/a	hspAfrica1WAfrica	Human Microbiome Project
GAM245Ai	West Africa	Gambia	n/a	hspAfrica1WAfrica	Human Microbiome Project
GAM260ASi	West Africa	Gambia	n/a	hspAfrica1WAfrica	Human Microbiome Project
GAM112Ai	West Africa	Gambia	n/a	hspAfrica1WAfrica	Human Microbiome Project
GAM115Ai	West Africa	Gambia	n/a	hspAfrica1WAfrica	Human Microbiome Project
GAM119Bi	West Africa	Gambia	n/a	hspAfrica1WAfrica	Human Microbiome Project
GAMchjs136i	West Africa	Gambia	n/a	hspAfrica1WAfrica	Human Microbiome Project
GAM246Ai	West Africa	Gambia	n/a	hspAfrica1WAfrica	Human Microbiome Project
GAM263BFi	West Africa	Gambia	n/a	hspAfrica1WAfrica	Human Microbiome Project
HP116Bi	West Africa	Gambia	n/a	hspAfrica1WAfrica	Human Microbiome Project
GAM121Aii	West Africa	Gambia	n/a	hspAfrica1WAfrica	Human Microbiome Project
GAM114Ai	West Africa	Gambia	n/a	hspAfrica1WAfrica	Human Microbiome Project
GAM80Ai	West Africa	Gambia	n/a	hspAfrica1WAfrica	Human Microbiome Project
GAM103Bi	West Africa	Gambia	n/a	hspAfrica1WAfrica	Human Microbiome Project
GAM249T	West Africa	Gambia	n/a	hspAfrica1WAfrica	Human Microbiome Project
GAM117Ai	West Africa	Gambia	n/a	hspAfrica1WAfrica	Human Microbiome Project
GAM105Ai	West Africa	Gambia	n/a	hspAfrica1WAfrica	Human Microbiome Project
GAM265BSii	West Africa	Gambia	n/a	hspAfrica1WAfrica	Human Microbiome Project
GAM254Ai	West Africa	Gambia	n/a	hspAfrica1WAfrica	Human Microbiome Project
GAM100Ai	West Africa	Gambia	n/a	hspAfrica1WAfrica	Human Microbiome Project
GAM270ASi	West Africa	Gambia	n/a	hspAfrica1WAfrica	Human Microbiome Project
GAM101Biv	West Africa	Gambia	n/a	hspAfrica1WAfrica	Human Microbiome Project
GAMchjs114i	West Africa	Gambia	n/a	hspAfrica1WAfrica	Human Microbiome Project
GAM239Bi	West Africa	Gambia	n/a	hspAfrica1WAfrica	Human Microbiome Project
GAM71Ai	West Africa	Gambia	n/a	hspAfrica1WAfrica	Human Microbiome Project
GAMchjs106B	West Africa	Gambia	n/a	hspAfrica1WAfrica	Human Microbiome Project
GAMchjs124i	West Africa	Gambia	n/a	hspAfrica1WAfrica	Human Microbiome Project
GAM231Ai	West Africa	Gambia	n/a	hspAfrica1WAfrica	Human Microbiome Project
GAMchjs117Ai	West Africa	Gambia	n/a	hspAfrica1WAfrica	Human Microbiome Project
GAM260BSi	West Africa	Gambia	n/a	hspAfrica1WAfrica	Human Microbiome Project
GAM96Ai	West Africa	Gambia	n/a	hspAfrica1WAfrica	Human Microbiome Project
GAM201Ai	West Africa	Gambia	n/a	hspAfrica1WAfrica	Human Microbiome Project
GAM244Ai	West Africa	Gambia	n/a	hspAfrica1WAfrica	Human Microbiome Project
GAM264Ai	West Africa	Gambia	n/a	hspAfrica1WAfrica	Human Microbiome Project
GAM83Bi	West Africa	Gambia	n/a	hspAfrica1WAfrica	Human Microbiome Project
SA29A	South Africa	South Africa	Ogies, Mpumalanga	hpAfrica2	Didelot et al. 2013
SA194A	South Africa	South Africa	Ogies, Mpumalanga	hpAfrica2	Didelot et al. 2013
SouthAfrica7	South Africa	South Africa	Soweto, Bantu origin	hpAfrica2	Duncan et al. 2013
SouthAfrica20	South Africa	South Africa	Soweto, Bantu origin	hpAfrica2	Duncan et al. 2013
SouthAfrica50	South Africa	South Africa	Soweto, Bantu origin	hpAfrica2	Duncan et al. 2013
SA144A	South Africa	South Africa	Ogies, Mpumalanga	hpAfrica2	Didelot et al. 2013
SA253A	South Africa	South Africa	Ogies, Mpumalanga	hpAfrica2	Didelot et al. 2013
SA160A	South Africa	South Africa	Ogies, Mpumalanga	hpAfrica2	Didelot et al. 2013
SA155A	South Africa	South Africa	Ogies, Mpumalanga	hpAfrica2	Didelot et al. 2013
SA47A	South Africa	South Africa	Ogies, Mpumalanga	hpAfrica2	Didelot et al. 2013
SA34A	South Africa	South Africa	Ogies, Mpumalanga	hpAfrica2	Didelot et al. 2013
SA175A	South Africa	South Africa	Ogies, Mpumalanga	hpAfrica2	Didelot et al. 2013
SA169C	South Africa	South Africa	Ogies, Mpumalanga	hpAfrica2	Didelot et al. 2013
SA233A	South Africa	South Africa	Ogies, Mpumalanga	hpAfrica2	Didelot et al. 2013

SA172C	South Africa	South Africa	Ogies, Mpumalanga	hpAfrica2	Didelot et al. 2013
SA166A	South Africa	South Africa	Ogies, Mpumalanga	hpAfrica2	Didelot et al. 2013
SA40A	South Africa	South Africa	Ogies, Mpumalanga	hpAfrica2	Didelot et al. 2013
SA36C	South Africa	South Africa	Ogies, Mpumalanga	hpAfrica2	Didelot et al. 2013
SA303C	South Africa	South Africa	Ogies, Mpumalanga	hpAfrica2	Didelot et al. 2013
UM054	Asia	Malaysia		hpAsia2	Kumar et al. 2014
NAB47	Asia	India	n/a	hpAsia2	-
UM067	Asia	Malaysia	Malaysian-Indian	hpAsia2	Rehvathy et al. 2013
FD423	Asia	Malaysia	Malaysian-Indian	hpAsia2	Rehvathy et al. 2013
UM084	Asia	Malaysia	Malay origin	hpAsia2	Rehvathy et al. 2013
UM114	Asia	Malaysia	Malaysian-Indian	hpAsia2	Rehvathy et al. 2013
UM018	Asia	Malaysia	Malaysian-Indian	hpAsia2	Kumar et al. 2014
FD430	Asia	Malaysia	Malaysian-Indian	hpAsia2	Rehvathy et al. 2013
FD703	Asia	Malaysia	Malay origin	hpAsia2	Rehvathy et al. 2013
Yangon190	Asia	Myanmar		hpAsia2	*
SA222A	Asia	South Africa	Ogies, Mpumalanga	hpAsia2	Didelot et al. 2013
FD535	Asia	Malaysia	Malaysian-Indian	hpAsia2	Rehvathy et al. 2013
Hp_TH2099					
NAK7	Asia	India	n/a	hpAsia2	-
SNT49	Asia	India	Santal tribe	hpAsia2	-
Yangon179	Asia	Myanmar		hpAsia2	*
Yangon233	Asia	Myanmar		hpAsia2	*
Myanmar52	Asia	Myanmar		hpAsia2	*
Myanmar51	Asia	Myanmar		hpAsia2	*
Yangon142	Asia	Myanmar		hpAsia2	*
Myanmar66	Asia	Myanmar		hpAsia2	*
Mandalay30	Asia	Myanmar		hpAsia2	*
Mandalay03	Asia	Myanmar		hpAsia2	*
Yangon202	Asia	Myanmar		hpAsia2	*
India7	Asia	India	n/a	hpAsia2	-
3754	Europe	France	Bordeaux	hpAsia3	*
NP05	Asia	Nepal	Nepal	hpAsia2	*
NP04	Asia	Nepal	Nepal	hpAsia2	*
NP05-107	Asia	Nepal	Nepal	hpAsia2	*
NP05-105	Asia	Nepal	Nepal	hpAsia2	*
NP05-278	Asia	Nepal	Nepal	hpAsia2	*
NP05-272	Asia	Nepal	Nepal	hpAsia2	*
NP05-250	Asia	Nepal	Nepal	hpAsia2	*
NP05-121	Asia	Nepal	Nepal	hpAsia2	*
NP05-112	Asia	Nepal	Nepal	hpAsia2	*
SSR4	Europe	Ireland	Dublin	hspEuropeN	*
SSR13	Europe	Ireland	Dublin	hspEuropeN	*
SSR8	Europe	Ireland	Dublin	hspEuropeN	*
26695	Europe	UK	n/a	hspEuropeN	Tomb et al. 1997
SSR14	Europe	Ireland	Dublin	hspEuropeN	*
HPKX_483_AG0C1	Europe	Sweden	Kalix, Norrland	hspEuropeN	Yannikis et al. 2008
SSR23	Europe	Ireland	Dublin	hspEuropeN	*
SSR40	Europe	Ireland	Dublin	hspEuropeN	*
SW7A	Europe	Wales	Swansea	hspEuropeN	*
SSR20	Europe	Ireland	Dublin	hspEuropeN	*
SW21A	Europe	Wales	Swansea	hspEuropeN	*
SSR22	Europe	Ireland	Dublin	hspEuropeN	*
HPAG1	Europe	Sweden	Kalix, Norrland	hspEuropeN	Oh et al. 2006
Lithuania75	Europe	Lithuania	n/a	hspEuropeN	-
SSR3	Europe	Ireland	Dublin	hspEuropeN	*
SSR2	Europe	Ireland	Dublin	hspEuropeN	*
SSR5	Europe	Ireland	Dublin	hspEuropeN	*
SSR7	Europe	Ireland	Dublin	hspEuropeN	*
SSR9	Europe	Ireland	Dublin	hspEuropeN	*
SSR33	Europe	Ireland	Dublin	hspEuropeN	*
SSR17	Europe	Ireland	Dublin	hspEuropeN	*
SSR43	Europe	Ireland	Dublin	hspEuropeN	*
BON 254	Europe	France	Bordeaux	hspEuropeN	*
SSR12	Europe	Ireland	Dublin	hspEuropeN	*
G27	Europe	Italy	n/a	hspEuropeN	Baltrus et al. 2009
3745	Europe	France	Bordeaux	hspEuropeN	*
B38	Europe	France	n/a	hspEuropeN	Thiberge et al. 2010
P12	Europe	Germany	n/a	hspEuropeN	Fischer et al. 2010
N6	Europe	France	n/a	hspEuropeN	*
3843	Europe	France	Bordeaux	hspEuropeN	*
SW23Ai	Europe	Wales	Swansea	hspEuropeN	*
PHI 092	Europe	France	Bordeaux	hspEuropeN	*

CHA 185	Europe	France	Bordeaux	hspEuropeN	*
3746	Europe	France	Bordeaux	hspEuropeN	*
OK113	East Asia	Japan	n/a	hpEastAsia	Yahara et al. 2013
52	East Asia	South Korea	n/a	hpEastAsia	-
XZ274	Asia	China	n/a	hpEastAsia	Guo et al. 2012
51	East Asia	South Korea	n/a	hpEastAsia	-
HLJHP193	Asia	China		hpEastAsia	-
F32	East Asia	Japan	n/a	hpEastAsia	Kawai et al. 2011
HLJHP253	Asia	China		hpEastAsia	-
HLJHP256	Asia	China		hpEastAsia	-
HLJHP271	Asia	China		hpEastAsia	-
Yangon244	Asia	Myanmar		hpEastAsia	*
Yangon222	Asia	Myanmar		hpEastAsia	*
OK310	East Asia	Japan	n/a	hpEastAsia	Yahara et al. 2013
CPY1962	East Asia	Japan	n/a	hpEastAsia	Blanchard et al. 2013
CPY6271	East Asia	Japan	n/a	hpEastAsia	Blanchard et al. 2013
83	East Asia	unknown	n/a	hpEastAsia	David Graham
CPY6261	East Asia	Japan	n/a	hpEastAsia	Blanchard et al. 2013
CPY1124	East Asia	Japan	n/a	hpEastAsia	Blanchard et al. 2013
CPY3281	East Asia	Japan	n/a	hpEastAsia	Blanchard et al. 2013
F30	East Asia	Japan	n/a	hpEastAsia	Kawai et al. 2011
Oct-98	East Asia	Japan	n/a	hpEastAsia	-
35A	East Asia	Japan	n/a	hpEastAsia	David Graham
F16	East Asia	Japan	n/a	hpEastAsia	Kawai et al. 2011
CPY1313	East Asia	Japan	n/a	hpEastAsia	Blanchard et al. 2013
F57	East Asia	Japan	n/a	hpEastAsia	Kawai et al. 2011
CPY6081	East Asia	Japan	n/a	hpEastAsia	Blanchard et al. 2013
CPY6311	East Asia	Japan	n/a	hpEastAsia	Blanchard et al. 2013
Yangon159	Asia	Myanmar		hpEastAsia	*
8A3	East Asia	unknown	Experimental	hpEastAsia	Graham 2004, Kenneman 2011
Manado-1	Asia	Indonesia	Manado	hpEastAsia	-
Taiwan-47	East Asia	Taiwan	n/a	hpEastAsia	-
UM077	Asia	Malaysia	Chinese Malay	hpEastAsia	Rehvathy et al. 2013
UM085	Asia	Malaysia	Chinese Malay	hpEastAsia	Rehvathy et al. 2013
UM111	Asia	Malaysia	Chinese Malay	hpEastAsia	Rehvathy et al. 2013
Mandalay38	Asia	Myanmar		hpEastAsia	*
UM007	Asia	Malaysia	Chinese Malay	hpEastAsia	Kumar et al. 2014
CPY1662	East Asia	Japan	n/a	hpEastAsia	-
Yangon173	Asia	Myanmar		hpEastAsia	*
FD506	Asia	Malaysia	Chinese Malay	hpEastAsia	Rehvathy et al. 2013
UM038	Asia	Malaysia	Chinese Malay	hpEastAsia	Rehvathy et al. 2013
UM032	Asia	Malaysia	?	hpEastAsia	Khosravi et al. 2013
UM023	Asia	Malaysia	Chinese Malay	hpEastAsia	Rehvathy et al. 2013
UM065	Asia	Malaysia	Chinese Malay	hpEastAsia	Rehvathy et al. 2013
UM066	Asia	Malaysia	Chinese Malay	hpEastAsia	-
UM034	Asia	Malaysia	Chinese Malay	hpEastAsia	Kumar et al. 2014
FD577	Asia	Malaysia	Chinese Malay	hpEastAsia	Rehvathy et al. 2013
FD568	Asia	Malaysia	Chinese Malay	hpEastAsia	Rehvathy et al. 2013
PeCan4	South America	Peru	n/a	Hybrid	-
Sat464	Ind. America	Peru	Satipo region	hspAmerind	-
Shi470	Ind. America	Peru	Shimaa village	hspAmerind	-
Shi169	Ind. America	Peru	Shimaa village	hspAmerind	-
Shi417	Ind. America	Peru	Shimaa village	hspAmerind	-
Cuz20	Ind. America	Peru	Cuzco	hspAmerind	-
Shi112	Ind. America	Peru	Shimaa village	hspAmerind	-
Aklavik117	North America	Canada	Aklavik	hspAmerind	-
v225d	South America	Venezuela	Piaroan indian	hspAmerind	-
Puno135	Ind. America	Peru	Puno	hspAmerind	-
Puno120	Ind. America	Peru	Puno	hspAmerind	-

References

1. Haas, R., Meyer, T.F., Van Putten, J.P.M. A flagellated mutants of Helicobacter pylori generated by genetic transformation of naturally competent strains using transposon shuttle mutagenesis. *Mol. Microbiol.* **8**, 753-760 (1993).
